# A simple optogenetic MAPK inhibitor design reveals resonance between transcription-regulating circuitry and temporally-encoded inputs

**DOI:** 10.1038/ncomms15017

**Published:** 2017-05-12

**Authors:** Raquel M. Melero-Fernandez de Mera, Li-Li Li, Arkadiusz Popinigis, Katryna Cisek, Minna Tuittila, Leena Yadav, Andrius Serva, Michael J. Courtney

**Affiliations:** 1 Molecular Signalling Laboratory, A.I. Virtanen Institute, University of Eastern Finland, Kuopio 70210, Finland; 2 Neuronal Signalling Laboratory, Turku Centre for Biotechnology, University of Turku and Åbo Akademi University, Turku 20520, Finland

## Abstract

Engineering light-sensitive protein regulators has been a tremendous multidisciplinary challenge. Optogenetic regulators of MAPKs, central nodes of cellular regulation, have not previously been described. Here we present OptoJNKi, a light-regulated JNK inhibitor based on the *As*LOV2 light-sensor domain using the ubiquitous FMN chromophore. OptoJNKi gene-transfer allows optogenetic applications, whereas protein delivery allows optopharmacology. Development of OptoJNKi suggests a design principle for other optically regulated inhibitors. From this, we generate Optop38i, which inhibits p38MAPK in intact illuminated cells. Neurons are known for interpreting temporally-encoded inputs via interplay between ion channels, membrane potential and intracellular calcium. However, the consequences of temporal variation of JNK-regulating trophic inputs, potentially resulting from synaptic activity and reversible cellular protrusions, on downstream targets are unknown. Using OptoJNKi, we reveal maximal regulation of c-Jun transactivation can occur at unexpectedly slow periodicities of inhibition depending on the inhibitor's subcellular location. This provides evidence for resonance in metazoan JNK-signalling circuits.

## Abstract (web summary)

Light-sensitive regulators of protein kinases could offer valuable insights into intracellular signalling. Here the authors design an optogenetic inhibitor of c-Jun N-terminal kinase (JNK) and show evidence for resonance in JNK signalling circuits in neurons, and use the same design principle to develop an inhibitor for p38MAPK.

Optogenetics is the application of synthetic genes combining light-sensitive moieties with effector domains that transduce light into changes of protein and cell function. Early implementations included light-driven proton pumps to charge mitochondria^1^ and regulation of G-proteins by exogenous rhodopsin to depolarize individually illuminated neurons^2^. Light-regulated synthetic channels are widely used in behavioural neuroscience^3^. Second-generation optogenetics^4^ transduces light into functional regulation of signalling proteins. This goes beyond regulated firing of neurons, allowing manipulation of specific signalling events with high spatiotemporal precision^5^,^6^. Multidisciplinary combinations of crystallography, directed evolution, molecular modelling, interweaving of homologous peptides and systematic iterative functional optimization^6^,^7^,^8^,^9^,^10^ have all been applied to develop optogenetic regulators of an increasing range of targets. Nevertheless, the number of optogenetically regulatable signalling targets remains limited. Optogenetic inhibitors are generated on a case-by-case basis and no generic guidelines exist for researchers wishing to develop optogenetic regulators of their proteins of interest.

We aimed to develop a reusable approach for optical actuators, using c-Jun N-terminal kinase (JNK) as a proof of concept. The original caged RacGTPase paradigm used light-induced unfolding of the Jα-helix of oat phototrophin1 LOV2 domain (*As*LOV2)^11^ to relieve steric hindrance^6^. However, matching hydrophobic patches on second light-oxygen-voltage (LOV2) and GTPase surfaces were required to confer photoregulation^6^ and steric hindrance alone was not sufficient. Engineering photoregulation into the related GTPase cdc42 necessitated crystallization of the LOV2-GTPase fusion protein^6^. An alternate strategy, developed to impose light-dependent steric hindrance to short effector peptides^8^,^9^, used sequences homologous to the Jα to be interwoven within the Jα sequence generating a chimera exhibiting light regulation^9^. This strategy is useful for sequences of sufficient Jα homology that their introduction prevents neither photoregulation nor peptide-target interaction. However, such interweaving can cause >10-fold increased photocycle rate^9^. Less homologous sequences were also embedded within or interwoven into the Jα-helix so that residues from Jα could be removed without loss of photoregulation in some cases, but not others^8^. In successful instances, this obviates the problematic need for engineering hydrophobic interactions into each fusion protein^6^. Similarly, a Jα homology strategy with multiple rational design/selection cycles led to a panel of 33 Jα-nuclear export sequence (NES) hybrids, of which two exhibited strong light regulation^10^. The group responsible also replaced Jα residues 540–546 with a 7-residue nuclear localization sequence (NLS) with less, but apparently sufficient, homology to generate light-regulated nuclear localization^12^,^13^. This overlap strategy has been effective on several occasions, but is challenging and seems unlikely to be feasible for peptides with no homology to Jα. Limitations remain unclear, however, as 5 of 30 bipartite NLS sequences tested, mostly fused to Jα.L546, also showed strong light regulation^12^, as did the photosensitive degron fused to *At*LOV2Jα residue P616 (ref. 14) (equivalent to *As*LOV2Jα.P547). Other optogenetic signalling regulators have been described but typically require two independent components and some even require exogenous chromophores^4^,^5^ unlike LOV2, a small (∼120 residue) photosensor domain based on a PAS (Per-Arnt-Sim domain) core that binds ubiquitous flavin mononucleotide (FMN) as chromophore^11^.

While trying to avoid these limitations with a LOV2Jα-based tandem bait-prey design, we unexpectedly observe effective photoregulated steric hindrance of the LOV2Jα domain on a fused competitor of JNK docking, JIP1 (JNK-interacting protein1, also known as MAPK8IP1) residues 157–167. From this, we derive a compact optogenetic JNK inhibitor, OptoJNKi. This can be generated in expression systems and introduced directly into cells, showing that recombinant optical regulators can be optopharmacological tools, complementing the optogenetic approach. Molecular dynamics simulation suggests that interaction of JIP inhibitor peptide C-terminal phenylalanine with a non-polar pocket between the LOV2 domain Aβ/Bβ loop and Iβ strand might stabilize the dark state, enhancing light regulation of JIP peptide availability. We derive from this a design strategy we implement for another mitogen-activated protein kinase (MAPK), p38. We fuse short peptides from the MKK3 D-domain sequence demonstrated to act as an unusually selective and potent inhibitor of p38 (ref. 15), followed by a C-terminal phenylalanine, to the *As*LOV2Jα domain to generate steric hindrance depending on the Jα peptide state. We find it possible to rapidly generate respectable dynamic range (difference in binding between dark-state and lit-state of two- to threefold at minimum) testing only a few peptides and demonstrate the corresponding Optop38i construct regulates p38MAPK in response to light. Maximal inhibition of neuronal JNK pathways by OptoJNKi occurs at unexpectedly slow and context-dependent illumination periodicities, rather than more frequent illumination. This suggests JNK pathways exhibit complex resonant properties which, when matched by corresponding input periodicities, leads to a larger effect. We propose our design framework could help rapidly generate optogenetic as well as optopharmacological inhibitors of new targets of interest, perhaps even beyond MAPKs.

## Results

### Optimization of lit-state/dark-state JNK-binding ratios

**Figure 1:**
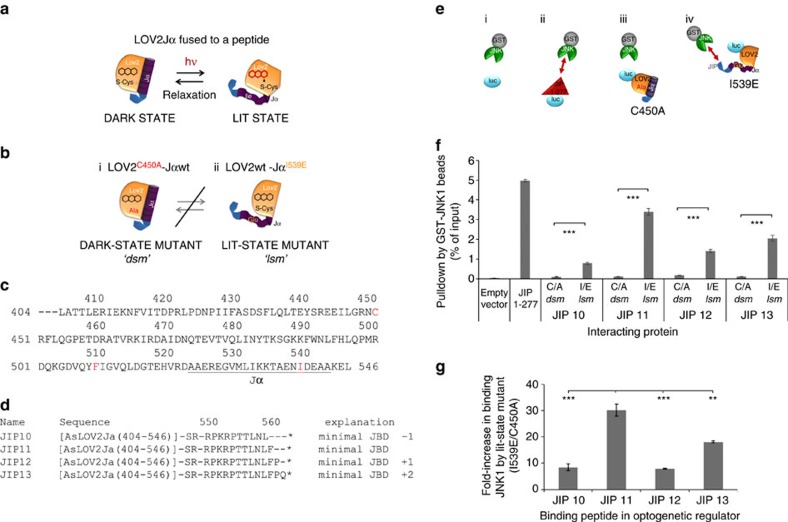
Optimization of lit-state/dark-state JNK-binding ratios. (**a**) Schematic representation of the LOV2 domain from plant phototrophin 1, which has been used to engineer light-regulated steric hindrance on fused peptides. Absorption of a photon by the FMN chromophore generates a covalent bond with a cysteine leading to unfolding of the Jα-helix and, ideally, increased accessibility of the peptide. (**b**) The mutationally stabilized forms of *Avena sativa* LOV2Jα are shown. (i) The ‘dark-state mutant' (*dsm*) harbours a C450A mutation that prevents covalent attachment of FMN, whereas (ii) in the ‘lit-state mutant' (*lsm*), the I539E mutant prevents Jα from forming a helix, increasing accessibility of the attached peptide. (**c**) The *As*LOV2Jα(404-546) sequence used is shown. Residues mutated for some experiments (cysteine 450 for dark-state mutant, isoleucine 539 for lit-state mutant and phenylalanine 509 for distal hydrophobic pocket disruption) are highlighted in red. (**d**) Sequence alignment of the 10–13 amino-acid JNK-inhibitory peptides and their fusion to *As*LOV2Jα (404–546) via XbaI linker (encoding a Ser–Arg dipeptide) is shown. (**e**) Schematic representation of pulldown assay using bead-immobilized GST-JNK1 incubated with luciferase-tagged constructs for quantification and normalization of inputs: (i) empty luciferase vector, (ii) the constitutive JBD JIP1-277 or (iii–iv) different JNK peptides fused to dark-state (C450A) or lit-state (I539E) mutants of *As*LOV2Jα. Pulldown efficiency is measured as the per cent of input luciferase signal on GST-JNK1 beads (normalized to input signal). (**f**) GST-JNK1 pulldown assay was performed using the inhibitory peptides as shown in Fig. 1d fused to LOV2Jα dark-state (C/A) or lit-state (I/E) mutants, with JIP1-277 as positive control and unfused (empty) luciferase vector as negative control. All peptides bind detectably to JNK1 but JIP1-277 exhibited the strongest binding followed by the lit-state mutant of LOV2Jα-JIP11 (*n*=3). (**g**) Dynamic range between lit and dark states was calculated for each JIP peptide based on the experiments shown in Fig. 1f. The LOV2Jα-JIP11, hereon referred to as OptoJNKi, shows the biggest change (*n*=3). Mean±s.e.m. is indicated; **indicates *P*<0.01, ***P<0.001. One-way ANOVA/Bonferroni post-test results are shown in Supplementary Data 1.

The FMN chromophore of *As*LOV2 absorbs light, leading to formation of a covalent bond with LOV2 residue C450 followed by unfolding of the Jα-helix. This process has potential to generate light-regulated peptides (Fig. 1a), but this has proven challenging to implement (see above). The *As*LOV2 C450A mutant prevents formation of the covalent bond and has been considered a dark-state mutant or *dsm* (Fig. 1bi; note this may not completely eliminate light responsiveness^16^), whereas I539E mutation in the Jα-helix permanently disrupts helix formation, mimicking the lit-state^6^,^17^,^18^,^19^ (*lsm*, Fig. 1bii). While evaluating strategies to circumvent limitations of previous optogenetic designs, we unexpectedly found that *As*LOV2 (404–546) (Fig. 1c) conferred lit/dark-state dependence of JNK binding to fused peptides. We compared fusion of *As*LOV2Jα mutants to 10–13 amino acids, corresponding to MAPK8IP1 sequences around 157–167 (refs 20, 21) (Fig. 1d). Equal amounts, tagged with firefly luciferase to facilitate quantitation^22^, were compared with JIP1-277, the N-terminal fragment of MAPK8IP1 that contains the JNK-binding domain (JBD)^21^, a constitutive inhibitor of JNK^21^,^23^,^24^. A washed pulldown assay with recombinant bead-immobilized GST-JNK1 (Fig. 1e) demonstrated that all peptides fused to LOV2Jα.*lsm* (I539E) bind to JNK1 (Fig. 1f). Positive control JIP1-277 exhibited the strongest binding followed by LOV2Jα.*lsm*-JIP11, which exhibited excellent dynamic range between lit and dark states (Fig. 1g). Therefore, we used *As*LOV2Jα-JIP11 fusion hereon for JNK inhibition, and refer to it as OptoJNKi after the cell-permeant D-form peptidic JNK inhibitor based on MAPK8IP1, D-JNKi^25^.

### MAPK-isoform specificity of OptoJNKi

**Figure 2:**
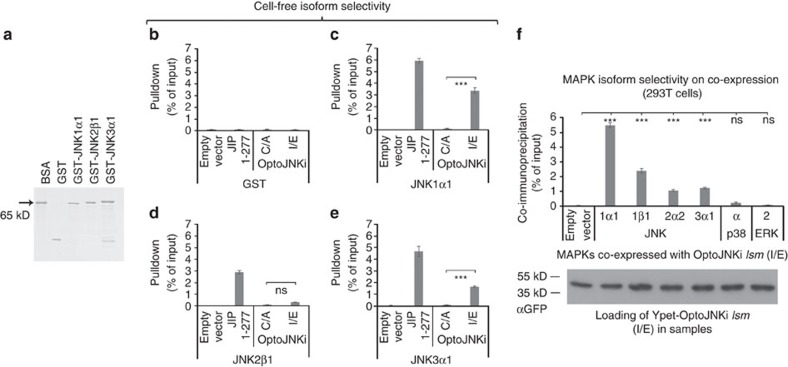
MAPK-isoform specificity of OptoJNKi. (**a**) Representative coomassie-stained SDS–PAGE gel of immobilized recombinant proteins used in this figure, calibrated against 0.25 μg BSA (65 kDa), are shown. (**b**–**e**) Pulldown assays (*n*=3) were performed using OptoJNKi LOV2Jα dark-state mutant (C/A), lit-state mutant (I/E), empty vector negative control, and JIP1-277 positive control. No binding was detected with GST (**b**). OptoJNKi.*lsm*, but not OptoJNKi.d*sm*, interacted with JNK1α1 and JNK3α1 (**c**,**e**) to about half the level as JIP 1-277. JIP 1-277 interacted less well with JNK2β1, which did not significantly bind either OptoJNKi variant. (**f**) OptoJNKi.*lsm* was co-expressed with luciferase-fused JNK isoforms, p38α or ERK2 in HEK293T cells to investigate selectivity of protein interaction in intact cells. Co-precipitated MAPK-isoforms were measured by luciferase activity on GFP-Trap_M microparticles after immunoprecipitation of OptoJNKi. OptoJNKi.*lsm* forms stable complexes with JNK1α1 and 1β1, and to a lesser extent JNK2α2 and JNK3α1, that is, representatives from each JNK gene and splicing type (α/β, long/short), but not with p38α or ERK2 (upper graph). Equal amount of precipitated OptoJNKi in each sample was confirmed by immunoblotting (*n*=3). Mean±s.e.m. is indicated; ns indicates not significant, ***P<0.001. One-way ANOVA/Bonferroni post-test results are shown in Supplementary Data 1.

Using purified recombinant GST-JNK isoforms (Fig. 2a) in cell-free conditions, we found interaction of OptoJNKi.*lsm* (I539E), but not OptoJNKi.d*sm* (C450A), with JNK1α1 and JNK3α1 (comparable to JIP1-277), but not with JNK2β1 nor with negative control glutathione *S*-transferase (GST) (Fig. 2b–e). We investigated complex formation in intact cells by co-immunoprecipitation. This showed that OptoJNKi.*lsm* forms stable complexes not only with JNK1α1, 1β1 and 3α1, but also JNK2α2, suggesting the construct is able to engage isoforms from all JNK genes (Fig. 2f). No interaction with p38α or ERK2 was detected.

### Spectrophotometric recording of light responsiveness

**Figure 3:**
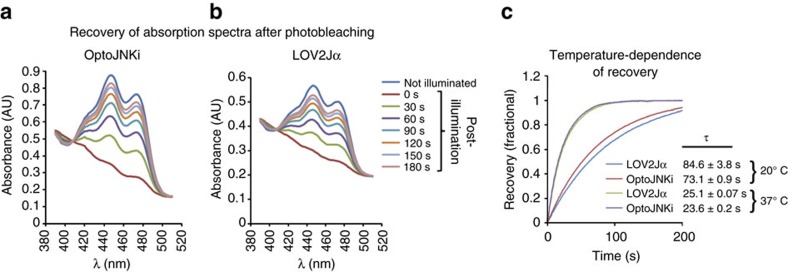
Photoswitching of LOV2Jα in OptoJNKi measured by spectrophotometry. (**a**) Absorption spectra of recombinant OptoJNKi (*As*LOV2Jα-JIP11, 1 mg ml^−1^), shown at 30 s intervals at 20 °C after bleaching (using 470 nm light, 6.4 mW cm^−2^ for 1 min), show a recovery consistent with FMN adduct decay with a *t*_½_ in the order of 60 s. Representative spectra are shown (*n*=3). (**b**) A corresponding absorbance spectral time series was acquired for recombinant *As*LOV2Jα (1 mg ml^−1^), showing high similarity with optoJNKi, suggesting minimal disturbance of the photocycle by fusion of the JIP11 peptide. Representative spectra are shown (*n*=3). (**c**) Absorbance recovery kinetics at 455 nm is shown for recombinant OptoJNKi and *As*LOV2Jα at both 20 and 37 °C. Traces are means of three replicates for each condition. Each individual replicate was fitted to exponentials and time constants, shown as mean±s.e.m. (*n*=3), indicate OptoJNKi has recovery kinetics marginally faster than *As*LOV2Jα. Strikingly however, adduct decay is >three times faster at 37 °C than 20 °C.

We generated an OptoJNKi construct without *lsm/dsm* mutations to evaluate photoswitching. LOV2 regulation is initiated by absorption of a photon by FMN, which usually enters a triplet state^26^,^27^ resulting in formation of an adduct with a conserved cysteine in the LOV domain^16^,^17^,^18^. This leads to the conformational change that unfolds the Jα-helix. The covalent bond between FMN atom C4a and cysteine is spontaneously broken with a time-constant typically ∼80 s depending on neighbouring amino acids and even on interweaving of peptides into Jα^9^. For practical applications, the relaxation rate of OptoJNKi from light switching should be determined. This can be achieved by monitoring the photocycle, that is, recovery of FMN absorbance after photobleaching (Fig. 3a). To determine the impact of JIP11 fusion, we compared the parental LOV2Jα protein (Fig. 3b). We found photocycles of OptoJNKi and LOV2Jα have time constants of 73.1±0.9 s and 84.6±3.8 s, respectively, at 20 °C (mean±s.e.m., *n*=3, Fig. 3c), consistent with literature values^26^ and suggesting the LOV2 photocycle of OptoJNKi is not greatly influenced by the JIP11 peptide, unlike the considerable impact of manipulations that integrate peptides within the Jα sequence^9^. However, temperature influences relaxation of some LOV2 domains^28^,^29^. As OptoJNKi was developed for use in mammalian cells, the >threefold faster adduct decay we measured at 37 °C (*τ*=23.6±0.2 s and 25.1±0.07 s for OptoJNKi and LOV2Jα, respectively; mean±s.e.m., *n*=3; Fig. 3c) is more relevant for cell-based applications.

### NLS-OptoJNKi inhibits endogenous c-Jun phosphorylation

**Figure 4:**
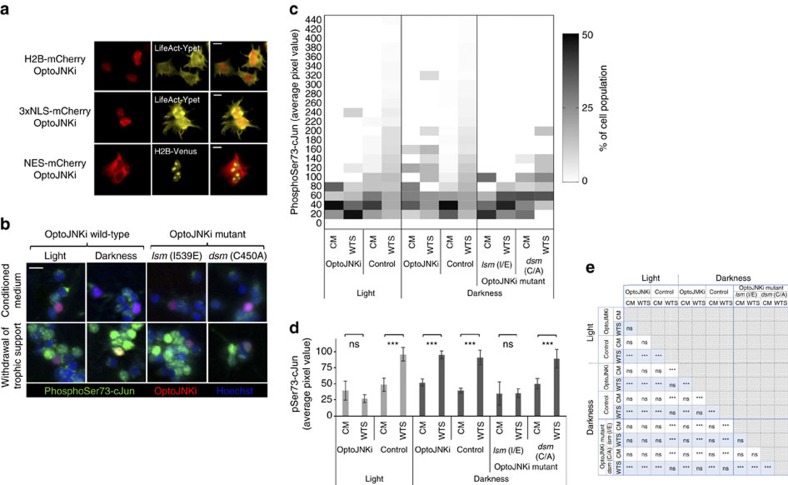
Gene transfer of OptoJNKi inhibits endogenous c-Jun phosphorylation in intact neuronal cells. (**a**) OptoJNKi is constrained to different subcellular compartments by fusion to targeting sequences H2B, 3 × NLS or NES together with mCherry to confirm localization visually in 293T cells with the assistance of cotransfected markers for F-actin (LifeAct-Ypet) or nucleus (H2B-Venus) as indicated. Scale bars, 10 μm. (**b**) Neuronal cultures immunostained for phosphorylation of JNK substrate c-Jun to measure the effect NLS-OptoJNKi on nuclear JNK signalling. WTS (lower panels) for 4 h increases nuclear p-Jun staining (green; DNA counter-stained in blue), when OptoJNKi-expressing cells (red) are kept dark or OptoJNKi.*dsm* is used (second and fourth column of panels). This is rare in illuminated or OptoJNKi.*lsm* expressing cells (first and third column), which show nuclear p-Jun responses predominantly in untransfected neighbour cells. CM controls show little nuclear p-Jun staining (upper panels). Scale bar, 10 μm. (**c**) The population distribution heat map of nuclear phospho-Jun responses of neurons shows minimal WTS-evoked pSer73-cJun response in OptoJNKi-transfected under white-light illumination. Quantified pJun immunostaining signal for each segmented nucleus positive for red fluorescence, an indicator of the mCherry-fused NLS-OptoJNKi variants used, was included under categories OptoJNKi, OptoJNKi.*lsm* or OptoJNKi.*dsm.* pJun signal from all mCherry-negative neighbouring cells was included in the control category. Most cells in CM have lower pSer73-cJun staining in all cases, whereas WTS shifts the population profile to higher p-Jun levels. The exceptions are the illumination/OptoJNKi (second column) and darkness/optoJNKi.*lsm* (10th column) conditions. (**d**) Average levels of pSer73-cJun per segmented nucleus are shown here for all conditions—with or without illumination, WTS and mCherry transfection marker. Only illumination of OptoJNKi-transfected cells led to complete suppression of the phospho-c-Jun response. Selected statistical comparisons from the complete analysis in Fig. 4e are shown above the bars to highlight significant changes induced by WTS (mean±s.e.m., *n*=3 or 9 in the case of untransfected controls). (**e**) Full statistical analysis of all conditions represented in Fig. 4d, by two-way ANOVA with Neuman–Keuls post-test, presented as a table where ns denotes non-significant, *P<0.05, **P< 0.01 and ***P<0.001.

We monitored the impact of OptoJNKi on phosphorylation of endogenous c-Jun, the best characterized JNK substrate. JNK substrates may reside in constrained subcellular loci—c-Jun is typically nuclear—and we reported the importance of designing accordingly localized inhibitors^23^. Thus, we fused OptoJNKi to NES, NLS or Histone2B (H2B) sequences as well as mCherry red fluorescent protein to localize the protein to cytoplasmic, nuclear and chromatin compartments, respectively (Fig. 4a). Withdrawal of trophic support (WTS) from neuronal populations strongly induces phosphorylation of c-Jun by JNK in the nucleus^24^,^30^. WTS generates a phospho-Jun response in about half the neurons (Fig. 4b; population distribution heat map in Fig. 4c). Transfection with OptoJNKi together with illumination strongly suppresses average phospho-Jun staining of neurons in cells (Fig. 4c,d, full statistical comparisons in Fig. 4e). Correspondingly, the lit-state mutant (*lsm*) suppressed the response, whereas the dark-state mutant (*dsm*) did not (Fig. 4c,d, four right-most columns).

### Recombinant OptoJNKi as an optopharmacological tool

**Figure 5:**
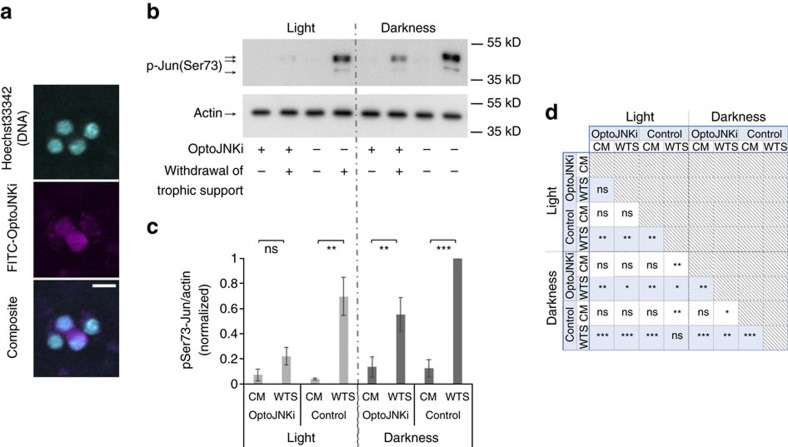
Recombinant OptoJNKi as an optopharmacological tool. (**a**) Cerebellar granule neuron cultures were incubated FITC-labelled His-TAT-OptoJNKi liposomes for 45 min. After 20 h, cells were fixed and DNA stained with Hoechst 33342. Fluorescein and Hoechst channels were imaged by spinning disk confocal microscopy, indicating successful delivery of protein into cells and partial presence in nuclear regions (individual false colour image channels—cyan for Hoechst and magenta for FITC—and merged channel are shown). Scale bar, 10 μm. (**b**) Recombinant optoJNKi protein was delivered into neuronal cell populations using a liposome method (as in Fig. 5a) where indicated. Neuronal cultures were subjected to WTS (4 h) or kept in CM as shown. In each case, cultures were illuminated (left lanes, 5 h starting one hour before withdrawal stress) or kept in darkness (right lanes). Phosphorylation of c-Jun was detected by immunoblot using lysate samples for each condition. A representative western blot is shown (*n*=3). (**c**) PhosphoSerine73-Jun levels from quantified films of replicates as shown in **b** were determined and normalized to actin (mean±s.e.m., *n*=3). Notably, only the combination of OptoJNKi delivery and continuous illumination provided complete suppression of p-Jun response. Selected statistical comparisons from the complete analysis in Fig. 5d are shown above the bars to highlight significant changes induced by WTS. (**d**) A full statistical analysis of all conditions represented in Fig. 5c, by two-way ANOVA with Neuman–Keuls post-test, are presented as a table. ns denotes non-significant, *P<0.05, **P< 0.01 and ***P<0.001.

Gene transfer is a common obstacle in optogenetics and may complicate efficient application to some cell types (for example, Fig. 4b). An optically regulated inhibitor that does not require DNA transfection, that is, an optopharmacological intervention can be more convenient in some cases. Optopharmacology is currently constrained to small molecule applications^31^ and has yet to be demonstrated with protein-based regulators. The transactivator of transcription (TAT) sequence from human immunodeficiency virus can efficiently deliver proteins into cells^22^,^25^,^32^,^33^, so we generated recombinant TAT-OptoJNKi. However, we observed no effects on cells with recombinant TAT-OptoJNKi, even if snap-misfolded by denaturation in urea followed by rapid removal, as recommended by standard TAT-loading protocols^32^. Therefore, we used lipid-based protein transfer^34^,^35^,^36^, retaining the TAT sequence as it has been reported to promote nuclear localization^37^. We observe partial nuclear localization of TAT-OptoJNKi loaded to cells with the lipid method (Fig. 5a), and immunoblotting showed almost complete suppression of c-Jun phosphorylation response by the combination of OptoJNKi and light (Fig. 5b,c; full statistical analysis presented in Fig. 5d), indicating recombinant OptoJNKi can be delivered to cells by this method.

### Regulation of ΔMEKK1-evoked GAL4-cJun by targeted OptoJNKi

Continuous white-light illumination showed that NLS-OptoJNKi can suppress phospho-Jun induced by 4 h WTS from *ex vivo* differentiated neurons (Figs 4 and 5), but we noted phototoxicity in pilot experiments longer than the 4 h window used. In Fig. 5, background effects of light alone are detected, the OptoJNKi levels used had some effect even in darkness, and the methods were noisy and time consuming. For a more efficient, precise and controlled approach to more accurately characterize and then apply OptoJNKi to cells, we used pulsed blue light-emitting diode (LED) illumination. We chose the GAL4-cJun reporter system as a more direct readout of JNK activity than endogenous c-Jun, which is not only phosphorylated by JNK but also transcriptionally induced by JNK-dependent and -independent pathways^24^,^38^. WTS is a valuable neuronal disease model, but it is an aggressive treatment that kills neurons and JNK/c-Jun activation kinetics are only known in cell populations, not in individual cells. These issues complicate characterization, so we first selected a more interpretable JNK-activating system by expressing a constitutive JNK-activating MAP3K.

**Figure 6:**
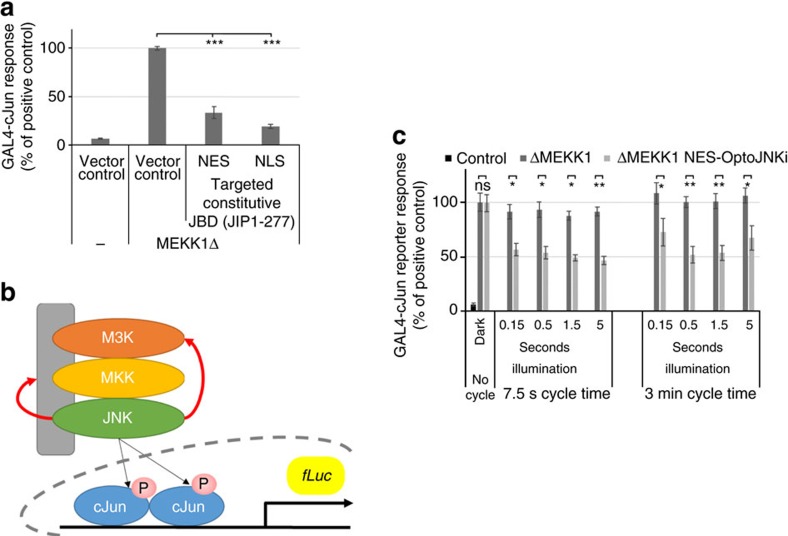
Regulation of ΔMEKK1-evoked GAL4-cJun by targeted OptoJNKi. (**a**) Cerebellar granule neurons transfected with GAL4-cJun reporter, pGL3-G5E4Δ38, RL-CMV, ΔMEKK1 (the constitutively active kinase domain of MEKK1) and either nuclear (NLS) or cytoplasmically targeted (NES) constitutive JBDs (residues 1–277 of JIP1 or MAPK8IP1) as shown. ΔMEKK1 induced strong GAL4-cJun activation, which was >50% inhibited by expression of constitutive JBD, whether targeted to nucleus or cytoplasm (*n*=12). (**b**) Schematic representation of activation of GAL4-cJun-driven luciferase reporter system by the JNK pathway after expression of constitutively active MAP3K. Red arrows indicate two of the multiple possible mechanisms that have been reported (feedback regulation of MAP3K^39^, feedback regulation of MAPK scaffold^40^) that could explain contributions of cytoplasmic JNK to GAL4-cJun activation using this system. (**c**) Cerebellar granule neurons transfected as in **a** but with NES-OptoJNKi receiving pulsed illumination (0.15–5 s duration, blue LED with 470 nm peak, at 0.6 mJ cm^−2^ s^−1^ representing a photon flux of 67 μmol m^−2^ s^−1^) every 7.5 s or 3 min as indicated. Illumination together with the presence of NES-OptoJNKi induced ∼50% inhibition of the response over this broad range of illumination conditions, but neither illumination alone nor NES-OptoJNKi alone had any effect (*n*=3). Mean±s.e.m. is indicated, ns indicates not significant, *P<0.05, **P<0.01, ***P<0.001. Analysis was carried out using by one-way ANOVA/Bonferroni and two-way ANOVA/Neuman–Keuls post-test (Supplementary Data 1).

ΔMEKK1, the constitutively active kinase domain of MEKK1 that is well tolerated by neurons at low-expression levels, induces ∼20-fold GAL4-cJun activation (Fig. 6a; without effect on global expression, see ‘Methods' section). This is inhibited over 50% by co-expression of JIP1-277, the constitutive JBD, whether it is NES or NLS targeted (Fig. 6a). This contrasts with WTS-induced GAL4-cJun activation sensitive only to NLS-JBD^23^ (Fig. 8b). Although GAL4-cJun contains multiple nuclear localization motifs, numerous cytoplasmic substrates of JNK have been reported that could explain additional sensitivity of MAP3K-activated GAL4-cJun to cytoplasmic JNK inhibitor^39^,^40^ (Fig. 6b). The incomplete inhibition may relate to activation of additional c-Jun kinases by ΔMEKK1 (ref. 41) or a contribution of JNK isoforms less sensitive to MAPK8IP1-based inhibitors^42^,^43^ (Fig. 2f).

We illuminated neurons expressing NES-OptoJNKi to determine the light sensitivity of the regulator in cells. We found ΔMEKK1-evoked GAL4-cJun could be inhibited up to 50% by NES-OptoJNKi over a wide range of illumination conditions (pulse durations from 0.15 to 5 s, with periodicities of either once per 7.5 s or once per 3 min) but it was not affected by NES-OptoJNKi in darkness (Fig. 6c). This suggests OptoJNKi is relatively straightforward to use, being responsive to a wide range of photon doses (10–330 μmol m^−2^=0.09−3 mJ cm^−2^, repeated every 7.5 or 180 s), which did not appear to adversely affect the neurons (no change in response in the absence of NES-OptoJNKi).

### Changing inhibitor cycle time reveals JNK pathway resonance

**Figure 7:**
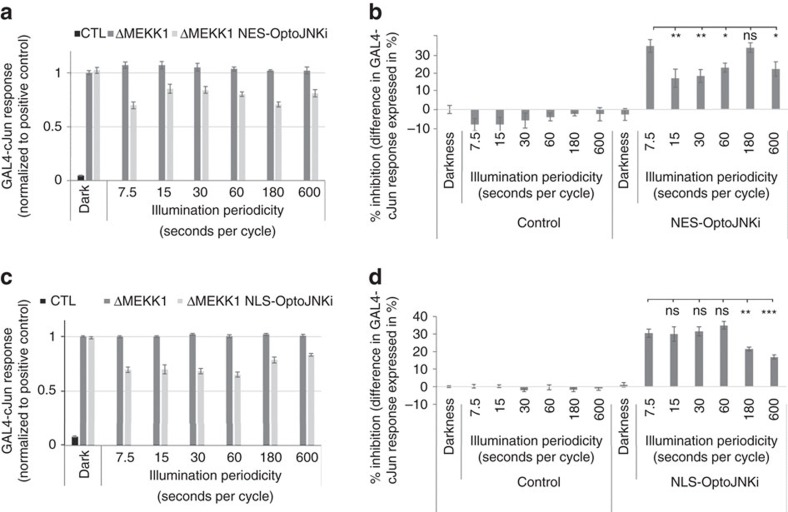
Changing periodicity of inhibition pulses reveals JNK pathway resonance. (**a**,**b**) Cerebellar granule neuron cultures were transfected with NES-OptoJNKi and the c-Jun reporter system as Fig. 6c. Wells were illuminated (1.5 s pulses at 600 μJ cm^−2^ s^−1^ as before) with periodicity as indicated. (**a**) The maximal inhibition of the ΔMEKK1-evoked GAL4-cJun response was achieved at 7.5 and 180 s periodicities. Less inhibition occurred between 7.5 and 180 s and inhibition falls once again after 180 s. (**b**) Percentage inhibition compared with ΔMEKK1/no OptoJNKi/darkness was calculated from the experiments presented in Fig. 7a. Significance of differences from 7.5 s periodicity in the presence of NES-OptoJNKi are highlighted (*n*=8). (**c**,**d**) Cerebellar granule neuron cultures were transfected and illuminated as in Fig. 7a,b but with NLS-OptoJNKi in place of NES-OptoJNKi. (**c**) Inhibition of the ΔMEKK1-evoked GAL4-cJun response was observed at 7.5–30 s periodicity but inhibition was stronger with 60 s periodicity, and fell at longer periodicities. (**d**) Percentage inhibition compared with ΔMEKK1/no OptoJNKi/darkness was calculated from the experiments presented in Fig. 7c. Significance of differences from 7.5 s periodicity in the presence of NLS-OptoJNKi are highlighted (*n*=8). Mean±s.e.m. is indicated, ns indicates not significant, *P<0.05, **P<0.01, ***P<0.001. Statistical analysis was carried out by two-way ANOVA/Neuman–Keuls post-test (Supplementary Data 1).

Optogenetic signalling regulators can be used to probe for unanticipated properties of cellular pathways that are otherwise difficult to observe directly, though they could have important impacts on cellular responses. Yeast MAPK pathways were only recently found to exhibit toxic MAPK resonance at a specific osmotic stress periodicity^44^ and we speculate that similar resonances might be identified with optogenetic tools such as OptoJNKi. Considering the 37 °C photocycle of recombinant OptoJNKi had a *t*_½_ ∼15 s (Fig. 3) and JIP peptides (affinity ∼800 nM, ref. 43) are not expected to form long-lived complexes with JNK, it was surprising that GAL4-cJun inhibition at 7.5 and 180 s periodicities were equal (Fig. 6c). Comparing different periodicities (Fig. 7a,b showing percentage inhibition calculated from Fig. 7a) reveals maximal inhibition at a periodicity of 7.5 s, twofold less inhibition at 15–60 s periodicity, but maximal inhibition once again at 180 s periodicity before falling again at 600 s periodicity. This quite unexpected behaviour suggests that, at 180 s periodicity, there is resonance with some aspect of JNK inhibition pathways, which leads to as strong overall inhibition as the most frequent illumination condition, even though the OptoJNKi was switched 12 times less often than at the less inhibitory 15 s periodicity.

NLS-OptoJNKi revealed no significant anomaly (Fig. 7c,d for percentage inhibition). Illumination periodicities from 7.5 to 30 s inhibit equally. At 60 s periodicity, one of the weakest conditions for NES-OptoJNKi (Fig. 7a,b) produces a marginally higher inhibition but not significantly so. Further lengthening periodicity is progressively less inhibitory (Fig. 7c,d). This suggests that the clear resonance anomaly occurs at specific periodicities in specific (cytoplasmic or nuclear) compartments, and relate to cellular signalling mechanisms in these compartments not to the common LOV2Jα-JIP11 component.

### JNK pathway resonance and critical activation periods

**Figure 8:**
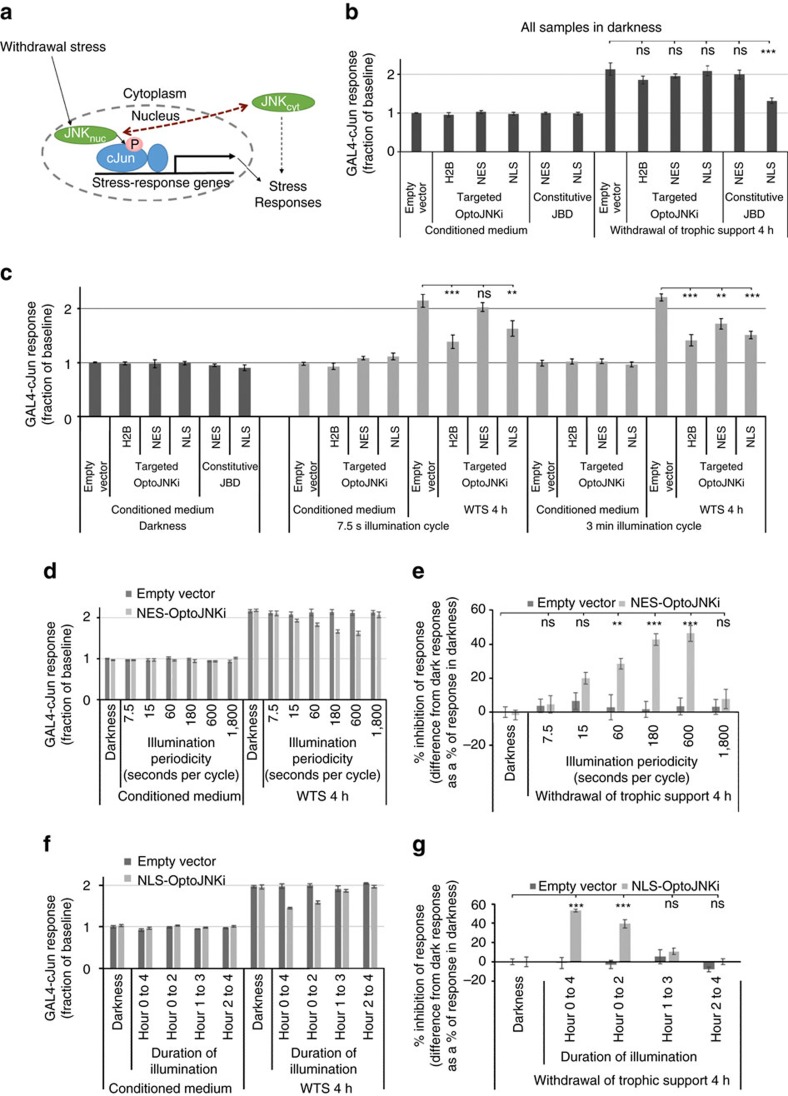
Resonance and critical activation periods during WTS-evoked JNK signalling. (**a**) Current understanding suggests cerebellar neuron WTS-evoked c-Jun involves nuclear pool of JNK, whereas cytoplasmic JNK was thought not to impact c-Jun regulation. However, data presented in this figure suggest that specific periodicities of cytoplasmic JNK inhibition does in fact lead to regulation of c-Jun transactivation (dotted red arrow). (**b**) Neurons expressing GAL4-cJun reporter (Figs 6 and 7) without ΔMEKK1 exhibit baseline GAL4-cJun signal without WTS (not phosphorylation-dependent because mutation of the sites has little effect^31^). Four hours WTS (20 h post-transfection) induces ∼twofold activation. In darkness, no targeted OptoJNKi had any effect. Constitutive JBD targeted to cytoplasm (NES) had no effect but NLS-constitutive JBD reduced the activation >50% as in previous reports^23^ (*n*=8). (**c**) Neurons as in Fig. 8b were illuminated as indicated (1.5 s pulses). H2B-OptoJNKi (targeted to chromatin) evoked strongest inhibition, followed by NLS-OptoJNKi. NES-OptoJNKi had no effect at 7.5 s periodicity but significantly suppressed at 3 min periodicity (*n*=8). (**d**,**e**) Neurons were transfected as in Fig. 8c showed increasing NES-OptoJNKi-dependent inhibition of 4 h WTS-evoked GAL4-cJun response over illumination periodicities from 7.5 to 600 s at which point inhibition was maximal. At 1,800 s periodicity, no inhibition was detected. (**e**) Percentage inhibition compared with the data normalization condition (CM/darkness/no OptoJNKi) from the experiments in Fig. 8d is shown and significance of differences from control are highlighted (*n*=4, or 6 for CM samples in darkness). (**f**,**g**) Neurons were illuminated by constant 1.5 s pulse per 7.5 s periodicity, either for the full 4 h, or only during the first two, second two or third two hours of a 4 h WTS period. (**g**) Percentage inhibition compared with the data normalization condition (CM/darkness/no OptoJNKi) from the experiments of Fig. 8f is shown. Statistical analysis indicates that inhibition was only obtained when illumination took place during the first hour and NLS-OptoJNKi was present (*n*=3). Mean±s.e.m. is shown; ns indicates not significant, *P<0.05, **P<0.01, ***P<0.001. Analysis was carried out using by two-way ANOVA/Bonferroni and Neuman–Keuls post-tests (Supplementary Data 1).

The WTS paradigm has been a useful model for general mechanisms of neurodegeneration and developmental neuronal death. Although JNK is generally thought to constitutively shuttle from cytoplasm and nucleus, in these neurons there is evidence, as summarized in the scheme (Fig. 8a), that under baseline conditions and during WTS (rather than after MAP3K overexpression, Fig. 6), cytoplasmic and nuclear pools are independent of one another^24^,^45^. First, JNK activity in neuronal cytoplasm is high and influences cytoskeletal and morphological parameters^23^,^45^,^46^,^47^, whereas nuclear JNK is more tightly coupled to stress responses such as WTS, leading to regulation of c-Jun^23^,^24^,^30^. This conclusion is also supported by effects of constitutive inhibitors of JNK as used in Fig. 6a, which has suggested that communication between cytoplasmic JNK and nuclear targets (red arrow) is minimal^23^,^46^.

GAL4-cJun remains a useful reporter under these conditions as it is activated relative to internal controls by WTS^24^ (note the reporter baseline without WTS is largely basal activity from dephospho-Jun because mutation of phosphorylation sites does not eliminate the baseline signal^24^). When neurons were maintained in darkness, expression of differentially targeted OptoJNKi and constitutive JBDs had no effect on baseline GAL4 activity nor did they affect the WTS response, with the sole exception of constitutive NLS-JBD (Fig. 8b), which is in line with a previous report^23^. Importantly, no targeted OptoJNKi had any effect in the dark.

When illuminated at 7.5 or 180 s periodicities, H2B-OptoJNKi most effectively suppressed the WTS response, followed by NLS-OptoJNKi with ∼50% inhibition of the WTS-induced response (Fig. 8c). NES-OptoJNKi was ineffective at 7.5 s periodicity; this should closely replicate the constitutive NES-JBD that is also ineffective (Fig. 8b). Surprisingly, at 180 s periodicity, NES-OptoJNKi was inhibitory. We explored the effect of NES-OptoJNKi on the WTS-evoked GAL4-cJun response in more detail. Again, NES-OptoJNKi had no effect at 7.5 and even 15 s periodicity of illumination, whereas inhibition increased as periodicity lengthened to 600 s before being lost at 1,800 s (Fig. 8d,e). This suggests that resonance also exists in the WTS-evoked JNK circuits, which may be more relevant to pathophysiological responses than ΔMEKK1-evoked GAL4-cJun.

It is known that WTS from cerebellar granule neurons for 4 h activates pools of JNK that regulate c-Jun and induce a GAL4-cJun response^24^,^30^,^48^, that c-Jun is increasingly phosphorylated and expressed during this period^24^ and that this leads to induction of pro-death factors and neuronal cell death^24^,^30^,^48^,^49^,^50^. Whether there is a specific critical period of JNK activation for c-Jun regulation during this 4-h period is unknown. Therefore, we transfected neurons with NLS-OptoJNKi and illuminated them for different time windows within the 4 h WTS. Illumination during the first 2 h inhibited the WTS-induced GAL4-cJun response ∼50%, but there was no effect on the response if the illumination was delayed by only 1 h (Fig. 8f,g). This suggests that the first hour of full JNK activation after WTS is necessary for the response measured at 4 h, and later partial inhibition has little effect.

### Molecular dynamics simulation predicts an interaction with a distal pocket

Fusing LOV2Jα to JIP11 (the minimal JNK-binding motif of MAPK8IP1) generated optimal lit/dark-state ratios (Fig. 1f,g) and light responsiveness (Figs 4, 5, 6, 7, 8). This was unexpected given previous observations that a specific hydrophobic interaction between effector and a Jα-proximal hydrophobic pocket on the PAS domain was necessary for the dark-state LOV2 to trap a fused effector^6^,^7^. However, the optimal JIP11 peptide terminated with a phenylalanine, providing a potential basis for hydrophobic trapping (Fig. 1d; note that JIP10 had no F but C-terminal L is also hydrophobic, whereas JIP12/13 still contained L and F close to the C terminus). Two independent crystallographic analyses showed phenylalanines, either from Switch-I of Rac1GTPase or from the C terminus of synthetic light-inducible dimers oLID/iLID, dock into the proximal hydrophobic pocket between LOV2 Bβ/Iβ strands that involves LOV2 residues I428, F429 and Y508 (refs 6, 7) in the packed crystal. Interestingly, the N-terminally trimmed photoregulated diaphanous autoinhibitor domain peptide fused to LOV2Jα residue 543 has a prominent internal phenylalanine at the C terminus of the inhibitory helix^51^. Addition of NDENYF peptide to the C terminus of a Jα with integrated SsrA peptide has been reported to improve dynamic range in response to light^9^. The light-regulated photosensitive degron has a phenylalanine one reside from its C terminus^14^ like JIP12. The light-regulated NES LEXY contains no F but terminates in leucine^10^ like JIP10. The seven optically regulated NLS constructs, including the bipartite ones, have V or L at the last or second last amino acid^12^.

However, it seemed unlikely that, in OptoJNKi, residue 10 or 11 of the JBD peptide could reach the same Jα-proximal hydrophobic pocket. In SsrA and iLid crystals, the C-terminal phenylalanine is only six amino acids beyond Jα residue A543 and it occupies the pocket in place of deleted Jα residue L546 (refs 7, 9). OptoJNKi retains Jα residue L546, which normally occupies the proximal Bβ/Iβ pocket^52^, and its C-terminal phenylalanine is 16 residues beyond Jα residue A543. To model the possible role of a C-terminal phenylalanine-PAS domain interaction in dark-state OptoJNKi in free solution, we carried out a molecular dynamic simulation based on pdb 2V1A^52^ (see ‘Methods' section).

**Figure 9:**
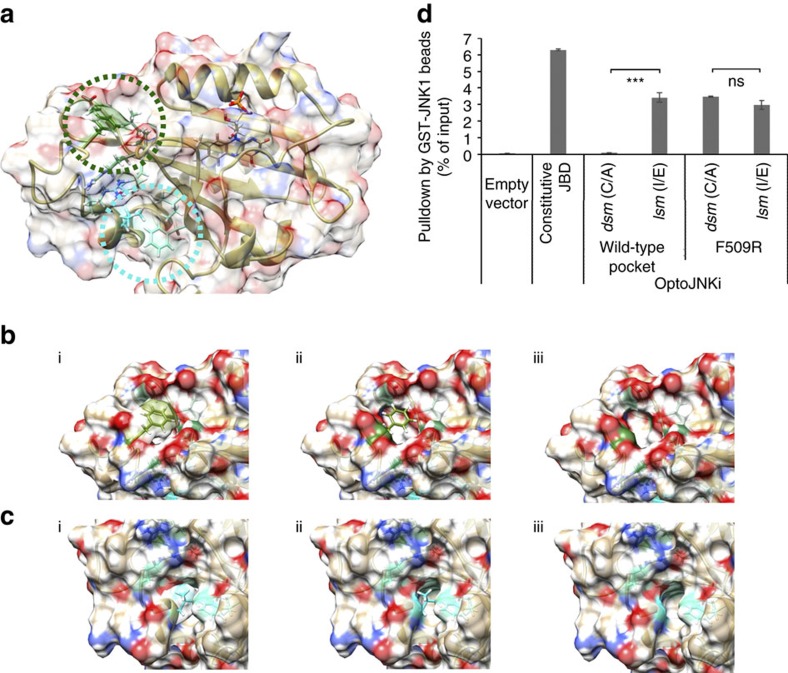
Molecular dynamics simulation of OptoJNKi suggests a potential photoregulation mechanism by hydrophobic tether capture in a new pocket. (**a**) Representation of the OptoJNKi structure deduced from time-averaged atom positions from a molecular dynamics simulation (see ‘Methods' section). The Cα chain is shown in tan. The structure is overlaid with a semi-transparent molecular surface. The atoms of F559 (the C terminus of the protein) and L546, together with interacting neighbouring residues, are highlighted green and cyan (carbon), respectively. Regions surrounding F559 and L546 are encircled. (**b**) The molecular packing of the terminal phenylalanine residue into a distal hydrophobic pocket in the OptoJNKi structure, as deduced by molecular dynamics simulation, is shown. Atoms shown are coloured tan (carbon), white (hydrogen), blue (nitrogen) and red (oxygen). F559 atoms are highlighted green (carbon), as are its interacting neighbouring residues, P420, R421, D505, V506 and F509. The overlaid semi-transparent molecular surface emphasizes (i) the hydrophobic pocket of the F559, (ii) the partial ‘caging' of F559 depicted in stick format and (iii) the depth of the hydrophobic pocket created by F559 (which is itself not shown in **b**iii to assist in visualization). (**c**) The corresponding molecular packing of the L546 residue into the Jα-proximal hydrophobic pocket in the optoJNKi structure as in **b** is shown. L546 atoms are highlighted cyan (carbon), as are its interacting neighbouring residues R549, Y508, I417 and F429. The overlaid semi-transparent molecular surface here emphasizes the hydrophobic pocket, full ‘caging' depth of the hydrophobic pocket created by L546 in (i)–(iii) as for F559 in Fig. 9b. (**d**) GST-JNK1 pulldown was performed as in Fig. 1 using the OptoJNKi (*dsm* and *lsm*) with a wide-type LOV2 pocket, the OptoJNKi (*dsm* and *lsm*) with LOV2 pocket mutant F509R, and the constitutive JBD (JIP1-277) as positive control. F509R mutants interacted with JNK1 similarly as wild-type OptoJNKi.*lsm* (I539E), failing to show any difference between lit- and dark-state mutants (*n*=3). Mean±s.e.m. is indicated, ns not significant, ***P<0.001. Analysis was carried out using by one-way ANOVA/Bonferroni post-test (Supplementary Data 1).

This simulation shows the C-terminal phenylalanine participating in van der Waals interactions with residues (coloured in forest green, Fig. 9a,b) P420, R421 (β/γ carbons, not the guanidinium), D505, V506, F509. These create a stable ‘packed' structure by ‘caging' the phenylalanine and shielding it from the solvent. L546 is more fully caged within LOV2 than F559 (C-terminal residue of OptoJNKi, Fig. 1d), and participates in van der Waals interactions with residues (in cyan, Fig. 9a,c) R549, Y508, I417 and F429 in the proximal hydrophobic pocket. Thus, our molecular dynamics simulation is consistent with OptoJNKi.L546 residing, as in wild-type *As*LOV2Jα^52^, in the proximal Bβ/Iβ pocket which is important for regulation of the peptides interwoven into the Jα-helix, peptides SsrAC and iLID^7^,^9^. The OptoJNKi C-terminal phenylalanine remains in a distal hydrophobic pocket involving Iβ- and A/B-loop residues over the entire simulation time (10 ns), suggesting this interaction constrains movement of the JBD peptide (Fig. 9a).

The distal hydrophobic pocket therefore represents a potential novel basis for photoreversible trapping of phenylalanines and similar residues near the C terminus, which could be used to render long peptides unavailable in the dark state. This predicts that a point mutation disrupting the hydrophobic pocket might limit lit/dark state differences. We replaced pocket residue F509 with arginine because its side chain starts with an aliphatic hydrocarbon chain (β/γ carbons), minimizing disruption of Lov2 structure, but is tipped with a hydrophilic guanidinium head group, maximizing disruption of the novel hydrophobic pocket. F509R does not affect interaction of JNK with OptoJNKi.*lsm* (I539E vs I539E.F509R), but now OptoJNKi.*dsm* (C450A.F509R) binds equally, in stark contrast to the parental OptoJNKi.*dsm* (C450A; Fig. 9d). This cannot be ascribed to trivial destabilization of the LOV2 domain, as F509R mutants express in soluble form to a similar level as the parental OptoJNKi forms.

The potential implication is that a simple *ab initio* design strategy might be devised for regulation of short inhibitor peptides by Jα folding and LOV2 photoswitching. In contrast to some of the demanding and labour-intensive design strategies published, our proposed strategy simply (i) selects an inhibitor peptide, (ii) terminates with a C-terminal phenylalanine, (iii) fuses the peptide to *As*LOV2 (L404- L546) followed by XbaI restriction site-encoded S-R dipeptide (Fig. 1c,d, eiv) and trims the peptide at N and C termini to about 10 amino acids to maximize differences between lit and dark states without loss of binding.

### Development of Optop38i

**Figure 10:**
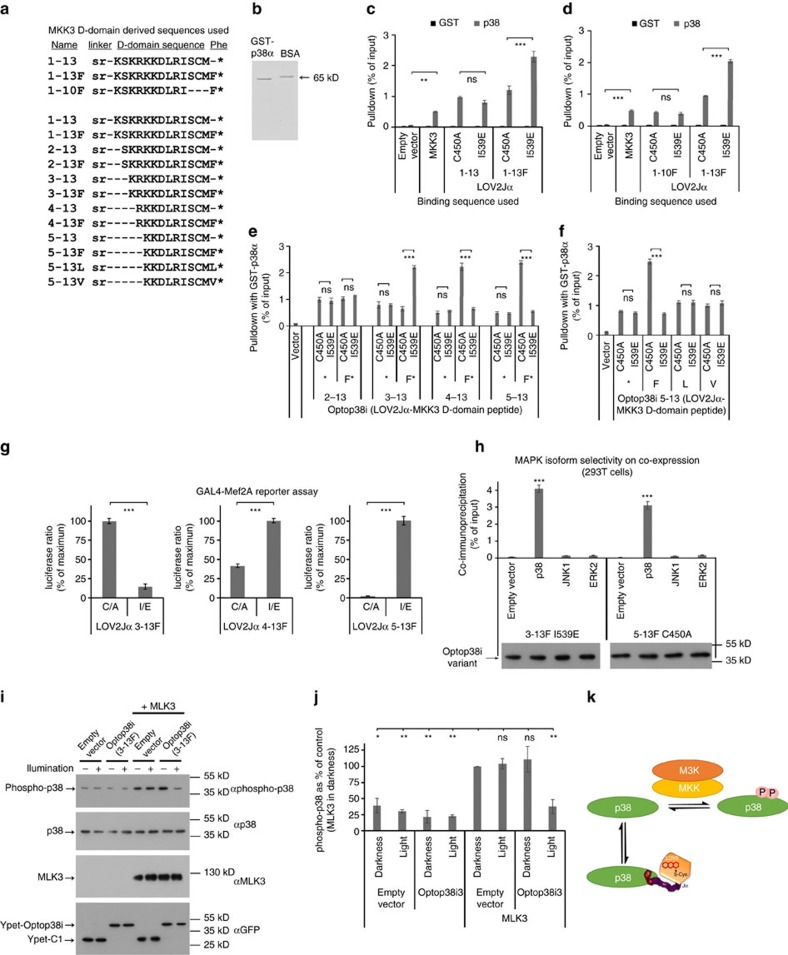
Optop38i design and functional validation. (**a**) Alignment of putative p38MAPK inhibitor sequences is shown. (**b**) Representative coomassie-stained gel of immobilized recombinant p38 calibrated against 0.25 μg BSA (65 kDa). (**c**) Luciferase-fused Optop38i 1–13 and 1–13F, *dsm* (C450A) and *lsm* (I539E), were evaluated by pulldown assay. All variants were efficiently pulled down by GST-p38, comparable to positive control Luc-MKK3b. Only addition of C-terminal phenylalanine resulted in significant change in pulldown between *dsm* and *lsm* (*n*=6 or 3 for 1–13). (**d**) Pulldown of luciferase-fused Optop38i 1–10F and 1–13F (*dsm* and *lsm*) was compared. Removal of C-terminal residues 11–13 eliminated *lsm*/*dsm* differences in pulldown (*n*=3). (**e**) N-terminal iterative trimming of inhibitor sequence with or without C-terminal phenylalanine was carried out to optimize *lsm*/*dsm* ratio. Optop38i 3–13F showed a larger *lsm*/*dsm* pulldown ratio than earlier variants. Shorter sequences 4–13F and 5–13F showed reversed *dsm*/*lsm* selectivity with the greatest pulldown ratio detect using 5–13F; removal of C-terminal phenylalanine eliminated *dsm*/*lsm* differences (*n*=3). (**f**) GST-p38 pulldowns with Optop38i 5–13, 5–13F, 1–13L or 5–13V showed that only Optop38i 5–13F exhibited LOV2Jα-state dependent binding (*n*=3). (**g**) Optop38i effects on p38-dependent transcription. Pairwise comparison of YFP-Optop38i *lsm* and *dsm* cotransfected 24 h with GAL4-Mef2A, p38 and constitutively active MKK3, produced lower reporter activity with Optop38i-state mutants showing stronger pulldown in Fig. 10e (3–13F.*lsm*, 4–13F.*dsm*, 5–13F.*dsm*). Statistical significance was evaluated by two-tailed *t*-test (*n*=3). (**h**) Optop38i 3–13F.*lsm* or 5–13F.*dsm* co-immunoprecipitated with p38MAPK but not JNK, ERK2 or vector. The representative immunoblots confirm equal Ypet-Optop38i expression (*n*=3). (**i**,**j**) 283T cells were transfected with MLK3 and Optop38i3 (wild-type 3–13F) or empty vectors for ∼20 h. Pulsed blue light was applied for 1 h. (**i**) Immunoblot of lysates show illumination of Optop38i3-expressing cutures inhibited MLK3-evoked phosphorylation of endogenous p38. Representative blots with loading control blots are shown. (**j**) Quantified replicates (*n*=3) and significance of differences are shown. (**k**) Scheme depicting inhibition of MLK3-evoked p38 phosphorylation by illuminated Optop38i3 as in figures. (**i**,**j**) Mean±s.e.m. is indicated, ns not significant, *P<0.05, **P<0.01, ***P<0.001. Statistical analysis was carried out by one- or two-way ANOVA and Bonferroni post-test where not otherwise stated (Supplementary Data 1).

To evaluate the design strategy, we selected p38MAPK as our target, considering its significance to cell function and human disease, and the lack of reported optical regulators. N-terminal peptides from MKK3 bind p38 (refs 53, 54) and block Mef2A phosphorylation by p38 in a highly selective and potent manner^15^. For the regulated p38 inhibitor (Optop38i), we began with a 13 amino-acid sequence (Fig. 10a, 1–13) conforming to the basic X_3-4_-ψxψ motif typical of D-domains^55^,^56^ with or without C-terminal phenylalanine. When fused with LOV2Jα, pulldown by immobilized recombinant GST-p38 (Fig. 10b) compares favourably with the p38 activator MKK3 (Fig. 10c). Importantly, pulldown was greater (∼twofold) in the lit state than in the dark state but only if C-terminal phenylalanine was present (Fig. 10c). C-terminal trimming to 10 amino acids resulted in reduced interaction but lost dependence on LOV2Jα mutational state even with C-terminal phenylalanine (Fig. 10d). Thus, even if a peptide retains competence to bind, additional requirements exist for engineering sensitivity to Jα state and the C-terminal phenylalanine is not sufficient. To optimize dynamic range, we further constrained the peptide by removal of amino acids from its N terminus. Considering the peptide may be partly helical, a single residue deletion could greatly influence orientation and in fact 2–13F loses responsiveness to LOV2Jα state (Fig. 10e). Responsiveness was recovered by removing one more amino acid with 3–13F, which enhanced dynamic range compared with 1–13F. Interestingly, the response to LOV2-Jα state was inverted by further deletion, generating constructs with high dynamic range, ∼fivefold with 5–13F, and high binding in dark state (Fig. 10e). We expect the shortest peptides may not reach the distal hydrophobic pocket of the PAS domain (Fig. 9a,b) unless Jα is unfolded, which may provide a rational explanation for the transition from lit-state binding to dark-state binding. Nevertheless, it is clear that our protocol in this case produced constructs sensitive to LOV2Jα state, improved dynamic range and moreover might provide a choice of active-to-inactive or inactive-to-active switching. To examine our strategy of adding C-terminal phenylalanine, we removed it from all constructs. In all cases, lit/dark state differences were lost (Fig. 10c,e). JIP10 has an alternative hydrophobic residue (leucine, Fig. 1d), reminiscent of some previously published peptides. Optop38i, however, is less tolerant as replacement of F with L or V eliminated lit/dark state differences (Fig. 10f).

We expressed the Optop38i panel (constructs 3–13F, 4–13F and 5–13F transferred to Ypet vectors) with a luciferase reporter system using p38-sensitive reporter GAL4-Mef2A (ref. 30), p38 and constitutively active MKK3. Consistent with the binding data, lit-state mutants show reduced p38 reporter activity using Optop38i (3–13F) and dark-state mutants show reduced activity in the case of Optop38i (4–13F)/(5–13F) (Fig. 10g). We evaluated MAPK selectivity in intact cells by immunoprecipitating Optop38i.*lsm* (3–13F) or Optop38i.*dsm* (5–13F) co-expressed with either p38α, JNK1 or ERK2 as in Fig. 2f. This demonstrated strong preference for the tagged-p38α over JNK1/ERK2 (Fig. 10h).

We used pulsed blue light to switch wild-type Optop38i in intact 293T cells. We have not found reliable phospho-specific antibodies for p38-specific substrates but D-domain motifs are used by MKKs to interact with their MAPK substrates, and D-domain peptides can also inhibit MAPK activation^53^,^54^. We therefore co-expressed Optop38i3 (wild-type *As*LOV2Jα fused to peptide 3–13F) with or without p38-pathway activator MLK3 (Fig. 10i,j). This shows that neither light nor Optop38i3 alone reduced MLK3-evoked p38 activation, but Optop38i3 allowed light-evoked regulation of p38 activation, presumably by competing with MKKs for p38MAPK (Fig. 10k).

## Discussion

The advent of optogenetics provided researchers with powerful tools that manipulate living systems with exquisite spatiotemporal precision using non-invasive visible light. The plethora of optical reporters to probe changes in cellular structure, second messenger levels, post-translational modifications and even conformation of individual proteins was until recently matched by few optical actuators, mostly chemically caged compounds released by ultraviolet light. Synthetic biology has now provided numerous designer channels and proteins that can be activated in different cells and distinct subcellular regions for precisely defined periods or using temporally patterned inputs. Nevertheless, the design of an optogenetic regulator for a new target is often a strenuous multidisciplinary task guided by combinations of crystallography, directed evolution, library screening, molecular modelling and/or mutational interweaving strategies^6^,^7^,^8^,^9^,^10^. It therefore remains challenging for a typical research lab to implement new optogenetic designs for new proteins and pathways.

Here we develop and evaluate a simple protocol for developing optogenetic regulators of new targets, specifically MAP kinases JNK and p38, for which the currently available tools are limited in spatiotemporal precision. We demonstrate that a LOV2-based regulator of JNK, OptoJNKi, can be applied as an optopharmacological tool without the need for gene transfer, and provide detailed protocols to successfully produce and transfer recombinant OptoJNKi into neurons. To target MAP kinases, we made use of known D-domain interactions^55^,^56^. We found *As*LOV2Jα sterically constrained JNK inhibitor peptides, showing considerable dark/lit-state JNK-binding ratios for the optimal peptide (∼30-fold, Fig. 1f,g). This was surprising considering reported difficulties achieving steric hindrance-based light regulation of either proteins or short peptides^6^,^8^,^9^. Molecular dynamic simulation suggested that phenylalanine at or near the C terminus could be ‘caged' by a novel non-polar LOV2 domain pocket formed between residues of the Iβ-sheet and the Aβ/Bβ loop (Fig. 9). This distal pocket might cooperate with the dark-state α-helical form of the Jα sequence to constrain and thereby generate steric hindrance of fused inhibitor peptide that can be released by light, leading to light regulation of JNK that we observed. In support of this, a point mutation in this pocket releases the JIP sequence (without disrupting protein stability as judged by soluble expression level) so that dark-state binding to JNK became indistinguishable from lit-state binding (Fig. 9d).

These structural considerations suggested a simple protocol to implement a novel light-regulated inhibitor peptide without modelling, interweaving or major experimental undertakings like crystallography and directed evolution. A known short inhibitor peptide is selected and fused to the C terminus of *As*LOV2Jα (404–546) and terminated by a phenylalanine. The binding of lit- and dark-state mutants, evaluated by pulldown with immobilized target, is optimized by systematic removal of C- and N-terminal residues from the peptide. Finally, selected constructs are validated in cell-based assays for known targets. This protocol was sufficient to engineer a several-fold difference between lit- and dark-state forms for interaction and inhibition of p38 in few steps without requiring modification of the LOV2Jα photosensor itself. We showed Optop38i3 allows a substantial light-evoked inhibition of MLK3-evoked p38 activation, though the constructs could perhaps be further optimized. Moreover, the isoform preference by OptoJNKi could potentially be refined to achieve fully isoform-selective optical regulation. Our optogenetic inhibitor design protocol might also be applicable to additional targets. Notably, D-domain selectivity can be reconfigured by minimal mutations^57^. Generation of new inhibitors at least for different MAPK family members may be straightforward.

To understand how important continuity of JNK activity is for traditional JNK outputs, we applied OptoJNKi in two JNK/c-Jun activation paradigms in primary cultured neurons. We found constitutive MAP3K-evoked transcriptional responses is inhibited by OptoJNKi upon illumination or by constitutive JNK inhibitor, whether localized to the nucleus or the cytoplasm (with NLS or NES motifs). In contrast to expectation, however, inhibition by OptoJNKi did not monotonically decrease as periodicity of brief (1.5 s) illumination pulses lengthened. NES-OptoJNKi exhibited maximal inhibition at relatively slow periodicities, showing sharply reduced inhibition in the 15–60 s periodicity range. The c-Jun activation response to 4 h WTS, a commonly used neurodegeneration paradigm^24^,^30^,^48^,^49^,^50^, was particularly dependent on the first hour of WTS, and once again it was most strongly blocked by a slow inhibition periodicity (1.5 s illumination every 10 min). These results are not consistent with a traditional kinase cascade model of the JNK pathway and indicate the presence of JNK-dependent regulatory pathways with distinct inhibitory resonances in a context and compartment-specific manner. It was recently reported that yeast MAPK pathways responding to osmotic stress resonate at a specific frequency of input stimulation, which results in substantial disruption of growth^44^. A corresponding MAPK resonance has not been reported in metazoans but the data presented here using periodic inhibition cycles may be the first evidence for this. Temporally encoded inputs are particularly relevant for neurons. Thus, mature neurons integrated into a network have synaptic neurotransmitter inputs known to regulate JNK and p38 pathways^58^,^59^,^60^, Moreover, cells like new-born neurons dynamically growing out and retracting processes most likely receive time-variant target-derived trophic support stimuli. These aspects have been very difficult to study, but tools like OptoJNKi and Optop38i may help achieve a more thorough understanding of how neuronal and other cells decode complex temporal and spatial patterns of MAPK pathway activation.

## Methods

### Plasmids including cloning and plasmid preparation

As a photosensor, we chose the LOV2 domain of *A. sativa* phototropin 1 (*As*LOV2)^11^. The LOV2Jα sequence used was taken from residues 404–546 of the light-activated histidine kinase NPH1 (nonphototropic hypocotyl 1). The native sequence was analysed for rare human codons (http://www.genscript.com/cgi-bin/tools/rare_codon_analysis) and rare codons were replaced to ensure neither tandem nor single rare codon usage, guanine-cytosine (GC) content ∼50%, ≤1 *cis* regulatory elements and no negative repeat elements. The advantages of this domain are that it is small, 110 amino acids plus 20 residues from the Jα-helix, it has a ubiquitous chromophore (FMN) and light induces a transient adduct and conformational change. The synthetic sequences were obtained by *de novo* synthesis (Mr. Gene, Regensburg, Germany).

We developed a vector encoding firefly luciferase fused to *As*LOV2Jα linked via a serine–arginine dipeptide (encoded by a XbaI site) to various MAPK8IP1-derived peptide sequences as indicated in the figures, to generate LOV2Jα-JIP10, 11, 12 and 13. These constructs contained LOV2Jα tandem as wild type or with a mutation known to stabilize the lit state (I539E, a mutation that prevents the α-helix from forming^18^) or the dark state (C450A, unable to form a covalent bind from the cysteine 450 to FMN so that absorption of light by FMN has been presumed to have no effect^17^. However, a recent report suggests this may not be completely eliminate light responsiveness^16^). We refer to the *As*LOV2Jα-JIP11 mutants as optoJNK.*lsm* and optoJNK.*dsm*, respectively. To facilitate reusability of this photoregulator scaffold, we included common unique restriction sites in the modular design as follows: NheI/AgeI-{luciferase or other tag}-BspEI/BglII/XhoI/EcoRI-{LOV2}-PstI-{Jα}-XbaI-{peptide}-BamHI. In addition, the PvuII site is a diagnostic site for constructs lacking the *dsm* mutation (C450A) in the LOV2 sequence and ClaI for constructs lacking *lsm* mutation (I539E) in Jα. In addition, mutation F509R was introduced to pLuc-OptoJNKi.*lsm* and OptoJNKi.*dsm* by polymerase chain reaction (PCR)-based procedures.

We transferred the OptoJNKi coding sequences (cds) in frame to plasmid vectors pmonoCherry-NES-C1 (nuclear export signal from MEK1), pmonoCherry-3xNLS-C1 (NLS, three copies of largeT-NLS) and pH2B-monoCherry (histone 2B). The former two vectors were prepared by replacing enhanced green fluorescent protein (EGFP) in the corresponding vectors that we developed previously, with cds for monomeric-Cherry. The cds was also inserted in frame into vector pYpet-C1 we prepared by replacing mCherry in pmCherry-C1 with the Ypet cds obtained from plasmid JNKAR1EV (generous gift of Michiyuki Matsuda, Kyoto, Japan). We also transferred OptoJNKi.*lsm*, OptoJNKi.*dsm* and JIP1-277 to a 3 × HA vectors for additional pulldown experiments, but pulldown of HA-JIP1-277 with recombinant JNK was less efficient than pulldown of luciferase JIP1-277 and the HA-tagged OptoJNKi forms were not stably expressed and could not be used. For recombinant expression and purification of the photoactivable protein, the cds for optoJNKi forms, and LOV2Jα (without JIP11) as a control, were subcloned in-frame after the His tag (6 × His) and TAT sequence (GRKKRRQRRR)^61^,^62^,^63^ in the vector pET28a-His-TAT, which we generated by inserting after the T7 tag sequence of pET28a a cds for TAT. All AsLOV2Jα/JIP sequences used are shown in Supplementary Fig. 1.

The Optop38i constructs with sequences as shown in Fig. 10 were generated by annealing and ligation of oligos (Macrogen, Korea) into the corresponding luciferase OptoJNKi plasmids from which JBD sequences were removed with XbaI and BamHI, and used for quantitative pulldown assays^22^,^33^. The entire Optop38i cassettes were transferred from pLuc to pYpet-C1 vectors to generate the panel for transcriptional reporter assays as well as co-immunoprecipitation experiments. All Optop38i sequences used are shown in Supplementary Fig. 5.

These constructs developed were sequenced to validate both inserts and ligation junctions. Full sequences and plasmids encoding the OptoJNKi and Optop38i constructs will be made available through addgene.org. The sequences for targeted OptoJNKi and the Optop38i3 forms (excluding the optional tags used) have been allocated GenBank accession numbers as follows: 3 × NLS-OptoJNKi (C/A), KY595935; 3 × NLS-OptoJNKi (I/E), KY595936; 3 × NLS-OptoJNKi, KY595937; NES-OptoJNKi (C/A), KY595938; NES-OptoJNKi (I/E), KY595939; NES-OptoJNKi, KY595940; OptoJNKi (C/A), KY595941; OptoJNKi (I/E), KY595942; OptoJNKi wild-type KY595943; Optop38i3 (C/A), KY595944; Optop38i3 (I/E), KY595945, Optop38i3 wild type, KY595946.

Plasmid p38α (human transcript variant 2) was cloned from 293T cDNA. JNKs 1α1, 1β1, 2α2, 2β1 and 3α1 were obtained by PCR from corresponding pcDNA3 plasmids (generous gift of Martin Dickens, University of Leicester) that we previously used for generating EGFP fusions^24^. ERK2 sequence was obtained from plasmid pECFP-ERK2 (ref. 64) (generous gift of Andrey Shaw, St. Louis, Missouri). These were transferred to pGEX-6P vectors (GE Healthcare) for production of GST fusion proteins and pLuc-C1 luciferase vector^32^ for co-immunoprecipitation experiments. The sequences of inserts were verified by Sanger sequencing (Macrogen).

Plasmids pLuc-MKK3b and pLuc-JIPJBD (residues 1–277) were prepared by cloning the human MKK3b isoform from 293T cDNA and transferring the JIP1 insert from plasmid pEGFP-JIP1JBD (residues 1–277), respectively, and transferring them in frame into firefly luciferase fusion vector pLuc-C1 (ref. 33). NES-JIP1-277 and NLS-JIP1-277 used in this paper were mKeima620 fusions, generated by transferring JIP1-277 into plasmids pmKeima620-NES and pmKeima620-3xNLS, that were themselves generated as above for Cherry-NES and 3 × NLS but using human-optimized cds for monomeric Keima620 (ref. 65) generated from PCR of overlapping oligos. pEBG-ΔMEKK1 was generated by inserting the rat MEKK1 kinase domain cds (encoding residues 1,174–1,493) to plasmid pEBG. Lifeact-Ypet was generated from pYpet vector by PCR-based procedures. pmonoCherry MKK3b-EE was derived from the MKK3b construct above by PCR-based methods. The pCGN-GAL4Mef2A (generous gift of Ron Prywes, Columbia University, New York), pGL3G5E4Δ38 (generous gift of Peter Shore, University of Nottingham), pcDNA3-GAL4cJun (5–105) (prepared by insertion of GAL4cJun cds to pcDNA3), pRL-CMV (Promega), H2B-Venus and H2B-Cherry plasmids (prepared by insertion of Venus and Cherry cds to pH2B-N1 vectors) and EGFP-hMLK3 (prepared by insertion of cds PCRed from HeLa cDNA into EGFP-C1) were the same as we have used in earlier studies^30^,^33^,^46^,^66^. All plasmids used in this study are listed in Supplementary Fig. 6.

### Cell culture and transfection

Cerebellar granule neuron cultures^67^ were prepared from postnatal day 7 rats (either sex). Cerebella were chopped in buffer B (PBS, 0.3% BSA, 14 mM D-glucose, 1.55 mM MgSO_4_) and incubated 15 min at 37 °C in buffer T (0.025% bovine trypsin in buffer B). Trypsinization was terminated by addition of 1/6th part buffer D (0.05 mg ml^−1^ Soybean trypsin inhibitor, 50 U ml^−1^ DNase, final 3.1 mM MgSO_4_ in buffer B). Trypsinized tissue was pelleted (1′, 100 *g*), resuspended in buffer D and mechanically dissociated by trituration with consecutive uses of 5 ml, 1 ml and 200 μl pipette tips. Resuspended cells were collected and pelleted (5′, 100 *g*) in buffer C (buffer B supplemented with 0.1 mM CaCl_2_ and final 2.8 mM MgSO_4_). All animal tissue isolation was carried out in accordance with national regulations and EU guidelines, under approval numbers EKS-003-2013 and EKS-003-2010 issued by the University of Eastern Finland Lab Animal Centre, and KEK/2015/2508 issued by the University of Turku Central Animal Laboratory. Cells were cultured in minimal essential medium (Gibco/Thermo Fisher, cat# 11700) supplemented with 10% (v/v) foetal bovine serum (Invitrogen), 33 mM glucose, 2 mM glutamine, 50 U ml^−1^ penicillin, 50 μg ml^−1^ streptomycin and 20 mM additional KCl. Cells were plated onto culture surfaces (either 12-well plates, 10 × 10 mm coverslips in 24-well plates or 96-well plates) coated with poly-L-lysine (15 μg ml^−1^). Culture medium was replaced after 24 h with the inclusion of 10 μM cytosine arabinofuranoside (Sigma) to reduce non-neuronal proliferation. Cells were cultured in a humidified 5% CO_2_ atmosphere at 37 °C.

For immunocytochemistry, cerebellar granule neurons were plated on 10 × 10 mm coverslips and they were transiently transfected at 5–6 days *in vitro* (DIV) in 24-well plates (total 2 μg DNA per well) with 85% Cherry NLS-OptoJNKi together with 15% of pCMV empty vector by the calcium phosphate method as follows^30^. The following is for a well in a 24-well plate and this is scaled up or down according to the number and area of culture surface to be transfected. Plasmid DNA (2 μg total) diluted to a final volume of 17.5 μl containing 1.25 μl 2.5 M CaCl_2_ is added dropwise to swirling 17.5 μl 2 × HeBS (1 × is 137 mM NaCl, 5 mM KCl, 0.7 mM Na2HPO4, 7.5 mM D-glucose, 21 mM HEPES, pH 7.06–7.14 selected batch-wise according to efficiency) in a polystyrene tube. The mixture is incubated in the dark 25' during which time a precipitate is formed, which is then added dropwise to the surface of media over neuronal cultures which have previously been washed and equilibrated 45 min in DMEM or MEM supplemented with 10 mM MgCl_2_. After a further 45 min incubation to allow Ca-DNA precipitates to reach the neuronal monolayer, the cultures are washed twice in DMEM or MEM-HCO_3_ (18 mM extra) supplemented with 10 mM MgCl_2_ and returned to conditioned media. For the reporter assays, cerebellar granule neurons were transfected at 6–9 DIV in 96-well plates by calcium phosphate method (total 0.4 μg DNA per well) with DNA combinations as described under reporter assays.

Human embryonic kidney 293T cells (293T, ATCC CRL-3216; not found in the ICLAC database of commonly misidentified lines and therefore not further authenticated but tested for mycoplasma negativity by DNA fluorochrome staining) were cultured in Dulbecco's minimal essential medium (with 10% foetal bovine serum, 19.4 mM supplementary glucose, 2 mM glutamine, 50 μg ml^−1^ streptomycin sulphate and 50 U ml^−1^ penicillin at 37 °C under 5% CO_2_. 293T cells were transfected with luciferase-fusion proteins for pulldown assays and fluorescent protein fusions for imaging. Transfections were performed with lipofectamine 2000 (Invitrogen) for imaging and for p38MAPK-pathway inhibition assay, and calcium phosphate method for protein expression for use in pulldown assays.

COS7 (ATCC CRL-1651; not found in the ICLAC database of commonly misidentified lines and therefore not further authenticated but tested for mycoplasma negativity by DNA fluorochrome staining) were cultured in minimal essential medium supplemented with 10% foetal bovine serum, 2 mM glutamine, 50 μg ml^−1^ streptomycin and 50 U ml^−1^ penicillin at 37 °C under 5% CO_2_. Transfection was performed with lipofectamine 2000 (Invitrogen) according to the manufacturer's instructions.

### Expression and purification of GST-fusion proteins for pulldowns

For expression of GST and GST-tagged JNK 1α1, 2β1, 3α1 and p38α, BL21 (DE3) *E. coli* cells transformed with expression vectors pGEX-6P JNK or p38, or pGEX-6P1 for GST, was used to inoculate in LB medium supplemented with 0.1 mg ml^−1^ ampicillin and 1% D-glucose (omitted for pGEX-6P1) and grown overnight in 37 °C. The inoculum was diluted 50-fold in fresh LB medium supplemented with 0.1 mg ml^−1^ ampicillin and 0.1% D-glucose and grown in 37 °C under continuous shaking (220 r.p.m.) until reaching OD_600_=0.5. Cells were induced to express protein by addition of final 250 μM isopropyl-beta-D-thiogalactopyranoside (1,000 μM for pGEX-6P1). The temperature was reduced to 23 °C (30 °C for pGEX-6P1) under continuous shaking (220 r.p.m.). After 18 h, cells were collected by centrifugation at 6,000*g* for 10 min at 4 °C. After one wash with PBS, the cells were collected.

For immobilization of GST proteins, the BL21 (DE3) cell pellet was resuspended in 10 ml per g wet weight of binding buffer (50 mM NaH_2_PO_4_/Na_2_HPO_4_ pH 8.0, 300 mM NaCl, 0.1% Triton X-100, 10% glycerol for the JNKs or PBS with 5% glycerol for p38α and GST, supplemented in all cases with 1 mM dithiothreitol, 0.2 mM phenylmethylsulphonyl fluoride (PMSF), 0.25 mg ml^−1^ DNase I (Applichem, 14,200 U mg^−1^) and lysed by vortexing with acid-washed glass beads (500–750 μm, Acros Organics) in 30 s cycles of vortexing and cooling on ice. Insoluble material was removed by centrifugation at 20,000*g*, 10 min at 4 °C and the soluble protein extract was collected, 0.2 μm filtered and cleared lysates were rotated overnight at 4 °C with Glutathione Sepharose 4 Fast Flow resin (GE Healthcare) pre-equilibrated with binding buffer. The beads were washed three times with 20 × bead bed volume of binding buffer (or PBS for GST) without DNase I to remove unbound proteins. The purity and amount of bound protein were calculated against BSA standards using coomassie-stained SDS–polyacrylamide gel electrophoresis (SDS–PAGE). Example gels are shown in the figures and full length lanes without cropping are shown in Supplementary Figs 2–4.

### Expression and purification of His-TAT-Lov2 fusions

For expression of His-TAT-OptoJNKi, BL21 (DE3) *E. coli* cells transformed with pET28a His-TAT-OptoJNKi plasmid were grown in 37 °C in medium supplemented with 0.03 mg ml^−1^ kanamycin and 0.1% D-glucose until reaching OD_600_=0.5. Cells were induced to express protein by addition of final 250–300 μM isopropyl-beta-D-thiogalactopyranoside. The temperature was reduced to 23–25 °C under continuous shaking (220–250 r.p.m.). Cells were collected by centrifugation for 10 min at 4 °C. After one wash with PBS, the cells were collected. Cell growth and protein expression were carried out as much as the procedure allowed in darkness (∼4 μW cm^−2^ measured), shielding samples (cultures, lysates, fractions, columns) from light with aluminium foil.

For purification of native His-TAT-OptoJNKi, it is usually recommended that TAT-fusions are prepared under denaturing conditions to facilitate passage across the plasma membrane^22^,^32^. For OptoJNKi, however, we compared a number of alternatives (see below) and only obtained satisfactory yields of soluble protein when we purified it under native conditions. For photocycle analysis, the pellet was resuspended in 10 ml per g wet weight of in lysis buffer (20 mM HEPES pH 8.0, 150 mM NaCl, 25 mM imidazole, 0.2 mM PMSF, 0.05% Triton X-100, 0.25 ml DNase I (Applichem, 14,200 U mg^−1^)) and suspended cells were lysed by vortexing with acid-washed glass beads (500–750 μm, Acros Organics) in 30 s cycles of vortexing and cooling on ice.

For cell-loading experiments, the pellet was resuspended in PBS pH 7.4, 0.2 μg ml^−1^ leupeptin, 0.2 μg ml^−1^ pepstatin, 0.2 μg ml^−1^ aprotonin, 50 μg ml^−1^ PMSF, 0.05% Triton X-100 and incubated with lysozyme (3 mg ml^−1^) at 4 °C for 30 min, followed by DNAse I (2.5 mg ml^−1^) plus 2.5 mM MgCl_2_ for 10 more minutes. In both cases, insoluble material was removed by centrifugation at 20,000*g*, 10 min at 4 °C, and the soluble protein extract was collected, 0.2 μm filtered and added to Ni sepharose 6 Fast Flow (GE Healthcare) with pre-equilibrated with immobilised metal ion affinity chromatography (IMAC) buffer A (20 mM HEPES pH 8.0, 150 mM NaCl, 25 mM imidazole) for photocycle analysis or the PBS lysis buffer supplemented with 10 mM imidazole for cell loading and rotated 2 h at 4 °C.

For photocycle analysis, the beads were washed twice with 10 × bead bed volume of IMAC buffer A followed by two times with 10 × bead bed volume of IMAC buffer A supplemented with 50 mM of imidazole, and protein was eluted in three washes of 2 × bead bed volume with IMAC buffer B (20 mM HEPES pH 8.0, 150 mM NaCl, 500 mM imidazole). For cell-loading experiments, the beads were washed twice with 10 mM imidazole in PBS, and eluted in PBS with increasing concentrations of imidazole (0.1, 0.25, 0.5 and 1 M). The purity and yield of the protein fractions were analysed by SDS–PAGE and selected fractions were desalted on a 5 ml HiTrap Desalting column (GE Healthcare). Protein concentration was determined by absorbance at 280 nm and DC protein assay (Bio-Rad). Fusion proteins were kept in the dark at 4 °C for short-term storage. Wild type and dark-state mutant proteins could be prepared successfully with this procedure but the lit-state mutant could only be obtained at very poor yield, possibly because the extended state of the Jα-helix reduces the solubility and stability of the fusion protein.

To obtain denatured His-tagged TAT-fused OptoJNKi, we modified the method of Becker-Hapak *et al*.^32^ The application of recombinant TAT-fusion proteins to cells has traditionally called for generation of snap-misfolded TAT-fusion protein to enhance cellular uptake followed by refolding in the cytoplasm^32^. Bacterial pellets from pET28a His-TAT-OptoJNKi cultures prepared as above were resuspended in buffer Z (8 M urea, 100 mM NaCl, 20 mM HEPES pH 8.0) and sonicated on ice for 1 min. Samples were then clarified by centrifugation at 16,000*g* for 10 min at 4 °C. Supernatant was equilibrated in 10 mM imidazole and Ni sepharose 6 Fast Flow was added. After rotation 1 h at room temperature, sepharose beads were collected into a spin column (Bio-Rad) and beads were washed with buffer Z plus 10 mM imidazole to remove nonspecific interactions. Then protein was eluted with increased concentrations of imidazole in Buffer Z (0.1, 0.25, 0.5 and 1 M). Concentration and purity was tested by SDS–PAGE gel and absorbance at 280 nm, indicating 1 M imidazole fractions contained the purest concentrated protein. To exchange the buffer to a non-denaturing environment that is compatible with cells, we performed the final elution from Ni sepharose beads with 1 M imidazole in PBS instead in buffer Z. Imidazole was then removed using a HiTrap Desalting column following the manufacturer's recommendations.

The main difficulty we found with this procedure is that unfolded protein precipitates after the original protocol's snap-misfolding step achieved by switching to a non-denaturing environment^32^. Buffer exchange by dialysis was not successful because it favoured precipitation of OptoJNKi. The alternative faster exchange of aqueous environment, using ion exchange chromatography was also not successful. When using source Q/S ionic exchange chromatography, as we have previously for His-tagged TAT-fusion proteins^32^, we found OptoJNKi precipitated on the column, presumably because of the hydrophobicity of the sequence. We succeeded to exchange buffer directly on the Ni sepharose beads as explained above. This produced higher yields of protein and reduced the number of steps for purification. All the procedures were performed near ‘darkness' (∼4 μW cm^−2^ measured)

### Conjugation of FITC to recombinant OptoJNKi

The protein was dialysed against 0.1 M NaHCO_3_/Na_2_CO_3_ pH 9.1 at 1,000-fold excess and concentration determined by DC protein assay (Bio-Rad). Fluorescein isothiocyanate (FITC, Invitrogen) was freshly dissolved in dimethylsulfoxide at a final concentration of 1 mg ml^−1^. FITC was added in five steps to a final concentration of 0.025 mg ml^-1^ to a 2 mg ml^−1^ protein solution. Labelling solution was kept dark and rotated 2 h at 4 °C, after which the labelling mixture was desalted using a 5 ml HiTrap desalting column pre-equilibrated with 20 mM HEPES pH 8.0, 150 mM NaCl before addition to cells.

### Protein interaction assays using the pulldown method

GST fusion proteins were prepared as described above, immobilized on glutathione-derivitized beads. Immobilized recombinant GST-fusion proteins (2 μg JNK and 10 μg p38 per assay) were incubated with cleared lysates of 293T cells expressing soluble proteins. Lysates were prepared using low-salt buffer^55^ (20 mM Na_2_ β-glycerophosphate, 30 mM NaF, 2 mM EDTA, 1 mM dithiothreitol supplemented with 0.2 mM Na_3_VO_4_ and protease inhibitors—10 μg ml^−1^ each leupeptin, pepstatin, aprotinin, 100 μg ml^−1^ PMSF and detergent 0.5% Igepal). Cell cultures were homogenized by pipetting and precleared by centrifugation for 5 min at 20,000*g* at +4 °C (ref. 33). When checked, luciferase activities in homogenates and cleared lysates were similar indicating a high level of solubility of expressed proteins. Lysates were normalized by activity to 7,000 units per assay (TD2020 luminometer, set at 50% sensitivity, 10 s integration time). Beads were rotated for 1 h at +4 °C to allow binding, and then they were washed three times and resuspended in CCLR (Promega) before quantification using luciferase assay reagent and data are shown as percentage stable binding. This is defined as 100 × (units of luciferase bound to the beads)/(units of luciferase in input). pLuc-C1 vector encoding only firefly luciferase and immobilized unfused GST were used as negative controls for comparison with luciferase fusions and GST-JNK/GST-p38, respectively.

### Protein interaction assays using the co-immunoprecipitation method

Co-immunoprecipitation of luciferase fusion proteins co-expressed with Ypet fusion proteins was performed as follows^22^,^33^. Luciferase-fused JNK isoforms, p38α or ERK2, were co-transfected with Ypet-OptoJNKi or Optop38i in 293T cells. Twenty-four hour after transfection, cells were lysed as above. Precleared lysates (16,000*g*, 10 min, +4 °C) were co-immunoprecipitated by rotating for 1 h at 4 °C with GFP-Trap_M microparticles (ChromoTek). Microparticles were captured with a tube rack equipped with permanent magnets and washed three times in the same buffer. Drained particles were resuspended in 60 μl CCLR (Promega), of which 20 μl was taken for immunoblotting to confirm even expression of OptoJNKi or Optop38i across samples, 20 μl for luciferase assay. Immunoblotting to detect the GFP variant Ypet that was used to tag the OptoJNKi and Optop38i constructs was carried out as described for GFP detection in the section ‘p38MAPK pathway inhibition assay' below. Luciferase signal were measured in resuspended particles as well as input samples. Precipitated activity was normalized to total levels for each sample as described above.

### Spectrophotometry

UV–vis spectra were acquired using a Jasco V-560 UV/VIS spectrometer installed with a temperature controller and sample holder PSC-498T and S (Jasco) with 1 nm bandwidth in a 1 cm path-length cuvette with a scan speed of 2,000 nm min^−1^ at 20 and 37 °C. Kinetic traces were acquired after photosaturation of the protein samples (1 mg ml^−1^ in PBS supplemented with 150 mM additional NaCl and 10% glycerol) using a 0.1 W blue LED for 60 s. Once the sample illumination was complete, it was scanned constantly. Samples were also measured at 455 nm with 1 nm bandwidth in a 1 cm path-length cuvette for 200 s after photosaturation at an interval of 0.5 s. Replicate scans were fit to a single exponential decay using the Microsoft Excel Solver Add-on. For spectrophotometry experiments, we compared adduct decay at 20 and 37 °C after photosaturation with either a 10 W white LED lamp (4,500 K colour temperature) or a 0.1 W blue LED (peak 465–467 nm). The power required to achieve comparable bleaching with the white LED and the blue LEDs was measured, using an Ex-Cite XR2100 power meter (LDGI), as 50 mW cm^−2^ (white LED) and 6.4 mW cm^−2^ (blue LED).

### GAL4-cJun luciferase reporter assays in cerebellar granule neurons

Cerebellar granule neurons were transfected with a firefly luciferase reporter plasmid driven by five GAL4 elements in tandem, pGL3-G5E4Δ38 (12.5% of total DNA), pcDNA3-GAL4cJun plasmid encoding the transactivation domain of c-Jun (residues 5–105) fused to the DNA-binding domain of GAL4 (residues 1–147; 12.5% of total DNA), and pRL-CMV (12.5% of total DNA) expressing sea pansy luciferase as an internal standard against which signals were normalized^24^. CMV was selected for normalization because pcDNA3-GAL4cJun is also driven by CMV promoter. To activate the JNK pathway, either pEBG-ΔMEKK1 (10% of total DNA), a plasmid encoding MAPK-kinase kinase 1 (MEKK1) kinase domain (amino acids 1,174–1,493) was co-transfected, or cells were subjected to 4 h of WTS. Differentially targeted mCherry-OptoJNKi constructs or empty vector (mCherry-C1) were co-transfected as indicated. For WTS experiments, all OptoJNKi constructs were transfected at 40% of total DNA. For the specific case of ΔMEKK1-co-expression experiments, mCherry-3xNLS-OptoJNKi was transfected at 40% of total DNA but pilot titration evaluations indicated light-independent artefacts at high levels of the NES-targeted form; by transfecting at only 2% of total DNA, we avoided effects in dark conditions, as can be seen in the figures. We did not find a suitable percentage level for use of H2B-OptoJNKi in ΔMEKK1-transfected cells, suggesting a possible unexpected interaction between these two constructs, so we were unable to use H2B-OptoJNKi in this specific JNK-activation model, even though there was no evidence of dark effects of H2B-OptoJNKi in the WTS model. This emphasizes the need for careful titration of constructs and inclusions of appropriate controls in every model. But this is not unique to OptoJNKi as the risks of overexpression are well known, potentially affecting use of ΔMEKK1 and most other constructs. It should be noted that, in cerebellar neuron cultures as used here, the 10% level of ΔMEKK1 plasmid used had little impact on overall expression as judged by Renilla luciferase internal control signal, but it substantially increased the GAL4-cJun-driven firefly luciferase response. For the ΔMEKK1 plates, 2 h after transfection, wells were individually illuminated as indicated. Illumination was carried out using blue LEDs (peak 465–467 nm) with 500 Hz pulsed-width modulation to achieve 0.6 mW cm^−2^ incident power on the well bottom (duty cycle modulation was set according to measured light at 100% duty cycle using an Ex-Cite XR2100 power meter from Lumen Dynamics/LDGI, Mississauga, Ontario, Canada). The illumination was delivered in pulses of 1.5 s length or as indicated. Pulses were repeated with periodicity and for time windows as indicated in the figures. For ΔMEKK1 experiments, dual luciferase reporter assay was carried out on samples lysed 18–20 h after transfection. For the WTS plates, conditioned cell culture medium was replaced by minimal essential medium (cat# 11700) 20 h after transfection and wells were maintained in a cell culture incubator (Cytomat 2C, Thermo) for a further 4 h before lysis, under the illumination conditions described. Dual luciferase reagent (Promega) was used to assay firefly (reporter) and Renilla (internal standard) luciferase activities in samples lysed in passive-lysis buffer (Promega) according to the manufacturer's instructions^24^.

### p38MAPK reporter luciferase assays in COS7 cells

Transcriptional activation of GAL4-Mef2A was assayed as follows^24^,^30^ using COS7 cells. Briefly, cells were transfected with pGL3-G5E4Δ38 (12.5% of total DNA), a plasmid expressing a fusion protein of the p38α-specific substrate Mef2A with the DNA-binding domain of GAL4 (12.5% of total DNA), and pRL-CMV (1% of total DNA). To activate the p38 pathway, a constitutively active MKK3 plasmid pmCherry-MKK3EE and pEBG-p38α (1% of the total DNA each) were co-transfected with individual Optop38i constructs (40% as indicated) as indicated together with pCMV (to equalize DNA amounts in all transfections). Twenty-four hour post transfection, cells were lysed and assayed as for the GAL4-cJun assay.

### p38MAPK pathway inhibition assay

293T cells were transfected with empty vector or wild-type Optop38i 3–13F (‘Optop38i3', 40% of total DNA), with or without the p38-pathway activator MLK3 (10% of total DNA) and pCMV (to equalize DNA amounts in all transfections), using lipofectamine 2000. Cell plates were illuminated 20–22 h after transfection with pulsed blue light (1.5 s pulses at 1.0 mW cm^−2^, 7.5 s periodicity) for 1 h. Cells were then rinsed twice with ice-cold PBS and lysed with 1 × Laemmli buffer (Tris-HCl 62.5 mM, pH 6.8, 1% SDS, 5% β-mercaptoethanol, 10% glycerol). Samples were collected and boiled at 95 °C for 10 min. Precleared cell lysates were analysed by western blotting. Control dark plates were covered with aluminium foil in the incubator and lysed in the same way as the lit plates. MLK3, wild-type Ypet-Optop38i3 and empty vector-derived Ypet expression levels were immunoblotted on nitrocellulose with antibodies raised against MLK3 (rabbit polyclonal C-20, Santa Cruz Biotechnology, cat# sc-536 RRID:AB_631936, at 0.2 μg ml^−1^) and GFP (Clontech Laboratories, Inc., cat# 632381, mouse monoclonal clone JL8, RRID:AB_2313808, at 0.1 μg ml^−1^). Endogenous p38MAPK activation-loop phosphorylation was measured by anti-phospho p38MAPK (rabbit polyclonal, Cell Signaling Technology, Beverley, MA; cat# 9211, RRID:AB_331641, at 1:1,000) and p38MAPK antibody (rabbit monoclonal, clone D13E1, Cell Signaling Technology, cat# 8690, RRID:AB_10999090, at 1:1,000) was used to detect total p38MAPK. Immunoreactivity was detected by corresponding HRP-labelled secondary antibodies (Santa Cruz Biotech, cat# sc-2004/sc-2005 at 1:25,000) and ECL detection (Pierce). Full films of blots are shown in Supplementary Fig. 4.

### Illumination of cells with white light

Cerebellar granule cell cultures at 6–7 DIV and transfected with Cherry-NLS OptoJNKi plasmid or OptoJNKi protein the day before were illuminated in a chamber (Okolab, with CO_2_ unit with active humidity control) or cell culture incubator (HeraCell, Heraeus, Hanau, Germany), respectively, with light generated from a 20 W white LED lamp (LED high power; 4,500 K, white). Cells at 37 °C were illuminated at a distance of 50 cm, which produced a continuous light intensity of 2.5 mW cm^−2^, measured with an X-Cite XR2100 power meter (LDGI). The illumination was started for 1 h in a cell culture incubator at 37 °C, 5% CO_2_, before WTS^24^,^30^). The cells were continuously illuminated during the WTS period of 4 h. Then, cells were fixed for immunostaining or lysed for western blot. Note that the power used to produce light switching in cell-free experiments at room temperature was 50 mW cm^−2^, which is more than that used on cells, as has been the case in previous studies^9^,^10^,^40^. This is most likely to be because cell-free switching experiments are carried out after a brief illumination period (bleaching is close to maximal in 5 s), but the white LED illumination of cells was continued for hours and a more gradual switching is sufficient, whereas excessive illumination can be toxic to the cells.

### Fluorescence labelling of neurons

Cerebellar granule neuron cultures on 10 × 10 mm coverslips were treated as indicated, washed in PBS and fixed with 4% paraformaldehyde for 20 min at room temperature. For immunostaining (Fig. 4), this was followed by permeabilization in 1% Triton X-100 phosphate-buffered saline for 3 min. Non-specific binding was blocked with 5% serum in 0.2% Tween-20/PBS for 1 h 20 min at room temperature. The coverslips were incubated with anti-P-c-Jun (Phospho-Serine73) rabbit monoclonal clone D47G9 (Cell Signaling Technologies) at 1:200 overnight at 4 °C followed washing and incubation with 1:500 anti-rabbit Alexa Fluor 488 (Invitrogen, cat# A-11034) for 1 h. In all cases, neurons were incubated with 1 μg ml^−1^ Hoechst 33342 in phosphate buffer to stain nuclei. For immunostaining and FITC-His-TAT-OptoJNKi localization, washed coverslips were mounted in DABCO-Mowiol mounting medium^67^.

### Fluorescence imaging and segmentation

All images shown were obtained with a BD855 pathway high-content image analyser. Images of Hoechst, Alexa488 and Cherry for Fig. 4 were acquired using a 40 × short-working distance water immersion objective (NA 1.15; Olympus) with appropriate filter combinations (using excitation and emission filters 380/10–435 nm long pass, 488/10–542/27 nm and 540/10 nm–580 LP, respectively). Multiple microscope fields were acquired per sample, and three independent samples were analysed for each condition. Transfected neurons were identified by fluorescence of monomeric Cherry protein that was fused to the OptoJNKi constructs. Data were analysed with AttoVision1.7 software (BD Biosciences) using automated background correction (constant percentage) of each channel followed by segmentation using Hoechst33342 images to seed polygons. Pre-processing parameters used only for the segmentation procedure were as follows: rolling ball 10 × 10, median 3 × 3, sharpen hat, Hoechst channel threshold 205–4,095 and to split cells close to each other, we used a watershed algorithm with an erosion of two pixels and object pixel size of 20–150 to exclude cells in clusters. Polygons drawn around the nuclei were manually reviewed to avoid the presence of incorrect selections and these were used to calculate mean pixel intensities in each nucleus from background-corrected raw Cherry and Alexa488 images. Cells with a threshold background-corrected value <4 in the Cherry channel were considered untransfected. This threshold value was chosen as follows—if the value is too high, few transfected cells are counted, generating noise (high s.d. between experiments); if the value is too low untransfected and transfected populations are inappropriately combined resulting in noise where the behaviour between populations is different. The optimal threshold is therefore the lowest value that did not increase noise in the data set. The neuronal population distribution of P-Jun levels under different conditions were presented by allocating the average P-Jun (Alexa488) pixel intensities per cell to bins of width 20 intensity units and graphing the results for Cherry-positive cells and Cherry-negative cells. The average and s.e.m. of c-Jun pixel intensities within segmented nuclei for each condition (lit vs dark, Cherry positive vs negative, CM vs WTS) was presented as a bar chart. For presentation of P-cJun levels (green channel) in Fig. 4b, example raw data was background subtracted with ImageJ (rolling-ball 250 pixels, smoothed paraboloid), the contrast for the green channel was set to identical levels (0–300) to facilitate comparison, and Hoechst/Cherry contrast was adjusted to the lowest levels in each panel so as to permit comparison of the green levels between cells and panels.

For localization of FITC-labelled His-TAT-OptoJNKi after liposome delivery (Fig. 5), FITC and Hoechst images were acquired with the BD855 pathway system using the same channels as above (380/10–435 nm long pass, 488/10–542/27 nm), but with the spinning disk engaged to acquire confocal images through the nuclei with a 40 × short-working distance air objective (NA 0.95; Olympus). As the FITC signal was very dim, exposure time was set at 5 s and background correction of acquired images were carried out with ImageJ settings as follows: 250 pixel radius rolling-ball/smoothed paraboloid.

### Protein delivery

Cerebellar granule neurons were plated on 12-well plates and the experiment was carried out after 5 DIV. The conditioned media was collected from the cells, and then they were washed twice with DMEM-10 mM MgCl_2_. The cultures were left in DMEM-MgCl_2_ and after 45 min the mix containing the protein (see below) was added. After 45 min, the cells were washed twice with DMEM-MgCl_2_ and conditioned media was added and the cells were keep in the incubator overnight before the experiment was carried out. For one well of a 12-well plate, 25 μl of DMEM, 1.21 μg of protein and 1.25 μl of ProteoJuice protein Transfection Reagent (Novagen) was used. The mix was left 20 min at room temperature and 225 μl of DMEM was added before the mix was dropped onto the cells. These conditions described above were also assayed without ProteoJuice to check if the TAT-fusion protein alone had any effect on the cells. This condition did not produce any detectable effect on cerebellar granule neuron cultures in a pilot experiment.

In a pilot experiment, we compared the Proteojuice reagent with the reagent SAINT/PhD (Synvolux Therapeutics, Leiden, the Netherlands). Both reagents appeared effective, but we selected Proteojuice for additional assays because the pilot indicated it had a larger effect. For the Synvolux reagent we used, for one well of 12-well plate, 2 μg of protein, 30 μl of HBS buffer (Synvolux) and 20 μl of PhD lipid (Synvolux). The mix was left 5 min at room temperature to allow the complex to form and 250 μl of DMEM was added before transferring the mix to the cell culture. The incubation of the cells with complex was carried out in conditioned media for 45 min, 3 h and overnight. The preliminary data indicated a larger effect with 45 min incubation.

### Immunoblotting of c-Jun regulation

Samples resolved on 10% SDS–PAGE were transferred by semidry transfer to polyvinyl difluoride. Primary antibodies, phosphoserine 73 c-Jun (rabbit monoclonal clone D47G9, Cell Signaling Technology, cat# 3270, RRID:AB_2129572) at 1:1,000 and β-actin (mouse monoclonal 2D1D10, GenScript, Piscataway, NJ; cat#A00702, RRID:AB_914102) at 0.2 μg ml^−1^ were used, followed by detection with corresponding HRP-labelled secondary antibodies (Santa Cruz Biotech, sc-2004/sc-2005 at 1:25,000) and ECL detection (Pierce). Full-length films of blots in Fig. 5 are shown in Supplementary Fig. 3.

### Molecular dynamics simulations

All simulations were carried out using a single crystal structure from the study of oat LOV2 involved in light-induced signal transduction (pdb ID 2V1A). The software package used for the simulations was the Desmond Molecular Dynamics System v2.2 (D.E. Shaw Research, New York, NY) and Schrodinger suite tools (Schrodinger, LLC, Portland, OR) and the calculations were performed on the CSC Finnish supercomputing clusters. Before the simulation, the crystal structure (2V1A) was prepared Schrodinger's Maestro tool by deleting crystallographic water molecules and assigning proper bond orders, and adding hydrogen atoms. The initial packing of the JIP11 peptide with the LOV2 PAS domain was determined using a high-resolution peptide-protein refinement implemented in the FlexPepDock tool. All molecular dynamics simulations were set up by surrounding the protein structure with *in silico* water molecules using the SPC water model in a solvation box of 10 Å, so that no protein atoms were outside of the water boundary. To approximate physiological conditions and system neutrality, Na^+^ and Cl^−^ ions (150 mM) were added to buffer the system. Desmond was used to perform all molecular simulations (OPLS-AA/2005 force field, NPT ensemble, 300 K, 1 bar and Berendsen coupling) with a multi-step minimization procedure using default settings to relax the system before simulation. For the molecular dynamics production runs, the NPT ensemble was used and a trajectory for 10 ns generated and visualized using the maestro trajectory player. Visualization images were created using University of California, San Francisco (UCSF) Chimera^68^ alpha version 1.5 (build 31329) software.

### Statistical analysis

For statistical analysis GraphPad-Prism was used, testing for normality whenever sufficient replicates were available. Data were analysed by one-way or two-way ANOVA with Bonferroni or Newman–Keuls multiple comparisons tests or by *t*-test (Fig. 10f). Resulting *P* values are shown in figure legends, *P*<0.05 being considered significant. *<0.05, **<0.01, ***<0.001. All data shown are means±s.e.m. with ns indicated, showing variation within each group are largely similar in any one bar chart. Full statistical analysis tables not shown within the figures (Figs 4 and 5) are included as Supplementary Data File 1. Sample size was selected based on pilot studies and to be able to evaluate statistical significance (*n*=3 minimum; *n* in all cases refers to biological replicates).

### Data availability

All relevant data are available from the authors on request.

## Additional information

**How to cite this article:** Melero-Fernandez de Mera, R.M.. *et al*. A simple optogenetic MAPK inhibitor design reveals resonance between transcription-regulating circuitry and temporally-encoded inputs. *Nat. Commun.* **8,** 15017 doi: 10.1038/ncomms15017 (2017).

**Publisher's note:** Springer Nature remains neutral with regard to jurisdictional claims in published maps and institutional affiliations.

## Supplementary Material

Supplementary Information Supplementary Figures and Supplementary References

Supplementary Data Full statistical analyses of data
