# Supplementary material for: A simple optogenetic MAPK inhibitor design reveals resonance between transcription-regulating circuitry and temporally-encoded inputs
Source: Nat Commun. 2017 May 12;8:15017. doi: 10.1038/ncomms15017 (PMC5437309; doi:10.1038/ncomms15017)
Supplement: Supplementary Information — Supplementary Figures and Supplementary References [file ncomms15017-s1.pdf]

Supplementary Figure 1: Amino acid sequences of AsLOV2Jα and fusions with JIP used with Clustal Omega[1] alignment.

>*As*LOV2Jα
LATTLERIEKNFVITDPRLPDNPIIFASDSFLQLTEYSREEILGRNCRFLQGPETDRATVRKIRDAIDNQTEVTVQLINYTKSGKKFWNLFHLQ
PMRDQKGDVQYFIGVQLDGTEHVRDAAEREGVMLIKKTAENIDEAAKEL*

>AsLOV2Jα-JIP11 = "OptoJNKi"
LATTLERIEKNFVITDPRLPDNPIIFASDSFLQLTEYSREEILGRNCRFLQGPETDRATVRKIRDAIDNQTEVTVQLINYTKSGKKFWNLFHLQ
PMRDQKGDVQYFIGVQLDGTEHVRDAAEREGVMLIKKTAENIDEAAKELSRRPKRPTTLNLF*

>AsLOV2Jα-JIP11.dsm(C450A)
LATTLERIEKNFVITDPRLPDNPIIFASDSFLQLTEYSREEILGRNARFLQGPETDRATVRKIRDAIDNQTEVTVQLINYTKSGKKFWNLFHLQ
PMRDQKGDVQYFIGVQLDGTEHVRDAAEREGVMLIKKTAENIDEAAKELSRRPKRPTTLNLF*

>AsLOV2Jα-JIP11.lsm(I539E)
LATTLERIEKNFVITDPRLPDNPIIFASDSFLQLTEYSREEILGRNCRFLQGPETDRATVRKIRDAIDNQTEVTVQLINYTKSGKKFWNLFHLQ
PMRDQKGDVQYFIGVQLDGTEHVRDAAEREGVMLIKKTAENEDEAAKELSRRPKRPTTLNLF*

>AsLOV2JαF509R-JIP11.dsm(C450A)
LATTLERIEKNFVITDPRLPDNPIIFASDSFLQLTEYSREEILGRNARFLQGPETDRATVRKIRDAIDNQTEVTVQLINYTKSGKKFWNLFHLQ
PMRDQKGDVQYRIGVQLDGTEHVRDAAEREGVMLIKKTAENIDEAAKELSRRPKRPTTLNLF*

>AsLOV2JαF509R-JIP11.lsm(I539E)
LATTLERIEKNFVITDPRLPDNPIIFASDSFLQLTEYSREEILGRNCRFLQGPETDRATVRKIRDAIDNQTEVTVQLINYTKSGKKFWNLFHLQ
PMRDQKGDVQYRIGVQLDGTEHVRDAAEREGVMLIKKTAENEDEAAKELSRRPKRPTTLNLF*

>AsLOV2Jα-JIP10.dsm(C450A)
LATTLERIEKNFVITDPRLPDNPIIFASDSFLQLTEYSREEILGRNARFLQGPETDRATVRKIRDAIDNQTEVTVQLINYTKSGKKFWNLFHLQ
PMRDQKGDVQYFIGVQLDGTEHVRDAAEREGVMLIKKTAENIDEAAKELSRRPKRPTTLNL*

>AsLOV2Jα-JIP10.lsm(I539E)
LATTLERIEKNFVITDPRLPDNPIIFASDSFLQLTEYSREEILGRNCRFLQGPETDRATVRKIRDAIDNQTEVTVQLINYTKSGKKFWNLFHLQ
PMRDQKGDVQYFIGVQLDGTEHVRDAAEREGVMLIKKTAENEDEAAKELSRRPKRPTTLNL*

>AsLOV2Jα-JIP12.dsm(C450A)
LATTLERIEKNFVITDPRLPDNPIIFASDSFLQLTEYSREEILGRNARFLQGPETDRATVRKIRDAIDNQTEVTVQLINYTKSGKKFWNLFHLQ
PMRDQKGDVQYFIGVQLDGTEHVRDAAEREGVMLIKKTAENIDEAAKELSRRPKRPTTLNLFP*

>AsLOV2Jα-JIP12.lsm(I539E)
LATTLERIEKNFVITDPRLPDNPIIFASDSFLQLTEYSREEILGRNCRFLQGPETDRATVRKIRDAIDNQTEVTVQLINYTKSGKKFWNLFHLQ
PMRDQKGDVQYFIGVQLDGTEHVRDAAEREGVMLIKKTAENEDEAAKELSRRPKRPTTLNLFP*

>AsLOV2Jα-JIP13.dsm(C450A)
LATTLERIEKNFVITDPRLPDNPIIFASDSFLQLTEYSREEILGRNARFLQGPETDRATVRKIRDAIDNQTEVTVQLINYTKSGKKFWNLFHLQ
PMRDQKGDVQYFIGVQLDGTEHVRDAAEREGVMLIKKTAENIDEAAKELSRRPKRPTTLNLFPQ*

>AsLOV2Jα-JIP13.lsm(I539E)
LATTLERIEKNFVITDPRLPDNPIIFASDSFLQLTEYSREEILGRNCRFLQGPETDRATVRKIRDAIDNQTEVTVQLINYTKSGKKFWNLFHLQ
PMRDQKGDVQYFIGVQLDGTEHVRDAAEREGVMLIKKTAENEDEAAKELSRRPKRPTTLNLFPQ*

```
CLUSTAL O(1.2.4) multiple sequence alignment


AsLOV2Jα                       LATTLERIEKNFVITDPRLPDNPIIFASDSFLQLTEYSREEILGRNCRFLQGPETDRATV
AsLOV2Jα-JIP11                 LATTLERIEKNFVITDPRLPDNPIIFASDSFLQLTEYSREEILGRNCRFLQGPETDRATV
AsLOV2Jα-JIP11.dsm(C450A)      LATTLERIEKNFVITDPRLPDNPIIFASDSFLQLTEYSREEILGRNARFLQGPETDRATV
AsLOV2Jα-JIP11.lsm(I539E)      LATTLERIEKNFVITDPRLPDNPIIFASDSFLQLTEYSREEILGRNCRFLQGPETDRATV
AsLOV2JαF509R-JIP11.dsm(C450A) LATTLERIEKNFVITDPRLPDNPIIFASDSFLQLTEYSREEILGRNARFLQGPETDRATV
AsLOV2JαF509R-JIP11.lsm(I539E) LATTLERIEKNFVITDPRLPDNPIIFASDSFLQLTEYSREEILGRNCRFLQGPETDRATV
AsLOV2Jα-JIP10.dsm(C450A)      LATTLERIEKNFVITDPRLPDNPIIFASDSFLQLTEYSREEILGRNARFLQGPETDRATV
AsLOV2Jα-JIP10.lsm(I539E)      LATTLERIEKNFVITDPRLPDNPIIFASDSFLQLTEYSREEILGRNCRFLQGPETDRATV
AsLOV2Jα-JIP12.dsm(C450A)      LATTLERIEKNFVITDPRLPDNPIIFASDSFLQLTEYSREEILGRNARFLQGPETDRATV
AsLOV2Jα-JIP12.lsm(I539E)      LATTLERIEKNFVITDPRLPDNPIIFASDSFLQLTEYSREEILGRNCRFLQGPETDRATV
AsLOV2Jα-JIP13.dsm(C450A)      LATTLERIEKNFVITDPRLPDNPIIFASDSFLQLTEYSREEILGRNARFLQGPETDRATV
AsLOV2Jα-JIP13.lsm(I539E)      LATTLERIEKNFVITDPRLPDNPIIFASDSFLQLTEYSREEILGRNCRFLQGPETDRATV
                               **********************************************.*************

AsLOV2Jα                       RKIRDAIDNQTEVTVQLINYTKSGKKFWNLFHLQPMRDQKGDVQYFIGVQLDGTEHVRDA
AsLOV2Jα-JIP11                 RKIRDAIDNQTEVTVQLINYTKSGKKFWNLFHLQPMRDQKGDVQYFIGVQLDGTEHVRDA
AsLOV2Jα-JIP11.dsm(C450A)      RKIRDAIDNQTEVTVQLINYTKSGKKFWNLFHLQPMRDQKGDVQYFIGVQLDGTEHVRDA
AsLOV2Jα-JIP11.lsm(I539E)      RKIRDAIDNQTEVTVQLINYTKSGKKFWNLFHLQPMRDQKGDVQYFIGVQLDGTEHVRDA
AsLOV2JαF509R-JIP11.dsm(C450A) RKIRDAIDNQTEVTVQLINYTKSGKKFWNLFHLQPMRDQKGDVQYRIGVQLDGTEHVRDA
AsLOV2JαF509R-JIP11.lsm(I539E) RKIRDAIDNQTEVTVQLINYTKSGKKFWNLFHLQPMRDQKGDVQYRIGVQLDGTEHVRDA
AsLOV2Jα-JIP10.dsm(C450A)      RKIRDAIDNQTEVTVQLINYTKSGKKFWNLFHLQPMRDQKGDVQYFIGVQLDGTEHVRDA
AsLOV2Jα-JIP10.lsm(I539E)      RKIRDAIDNQTEVTVQLINYTKSGKKFWNLFHLQPMRDQKGDVQYFIGVQLDGTEHVRDA
AsLOV2Jα-JIP12.dsm(C450A)      RKIRDAIDNQTEVTVQLINYTKSGKKFWNLFHLQPMRDQKGDVQYFIGVQLDGTEHVRDA
AsLOV2Jα-JIP12.lsm(I539E)      RKIRDAIDNQTEVTVQLINYTKSGKKFWNLFHLQPMRDQKGDVQYFIGVQLDGTEHVRDA
AsLOV2Jα-JIP13.dsm(C450A)      RKIRDAIDNQTEVTVQLINYTKSGKKFWNLFHLQPMRDQKGDVQYFIGVQLDGTEHVRDA
AsLOV2Jα-JIP13.lsm(I539E)      RKIRDAIDNQTEVTVQLINYTKSGKKFWNLFHLQPMRDQKGDVQYFIGVQLDGTEHVRDA
                               ********************************************* **************

AsLOV2Jα                       AEREGVMLIKKTAENIDEAAKEL*---------------
AsLOV2Jα-JIP11                 AEREGVMLIKKTAENIDEAAKELSRRPKRPTTLNLF*--
AsLOV2Jα-JIP11.dsm(C450A)      AEREGVMLIKKTAENIDEAAKELSRRPKRPTTLNLF*--
AsLOV2Jα-JIP11.lsm(I539E)      AEREGVMLIKKTAENEDEAAKELSRRPKRPTTLNLF*--
AsLOV2JαF509R-JIP11.dsm(C450A) AEREGVMLIKKTAENIDEAAKELSRRPKRPTTLNLF*--
AsLOV2JαF509R-JIP11.lsm(I539E) AEREGVMLIKKTAENEDEAAKELSRRPKRPTTLNLF*--
AsLOV2Jα-JIP10.dsm(C450A)      AEREGVMLIKKTAENIDEAAKELSRRPKRPTTLNL*---
AsLOV2Jα-JIP10.lsm(I539E)      AEREGVMLIKKTAENEDEAAKELSRRPKRPTTLNL*---
AsLOV2Jα-JIP12.dsm(C450A)      AEREGVMLIKKTAENIDEAAKELSRRPKRPTTLNLFP*-
AsLOV2Jα-JIP12.lsm(I539E)      AEREGVMLIKKTAENEDEAAKELSRRPKRPTTLNLFP*-
AsLOV2Jα-JIP13.dsm(C450A)      AEREGVMLIKKTAENIDEAAKELSRRPKRPTTLNLFPQ*
AsLOV2Jα-JIP13.lsm(I539E)      AEREGVMLIKKTAENEDEAAKELSRRPKRPTTLNLFPQ*
                               *************** *******
```

Supplementary Figure 2: Whole gel and film lengths for images in Fig. 2

A.

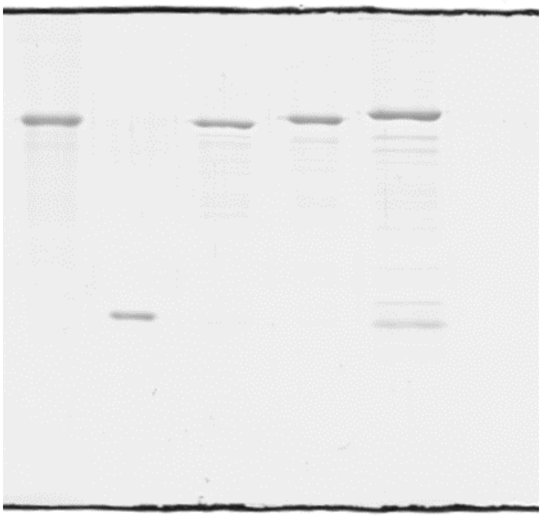

B.

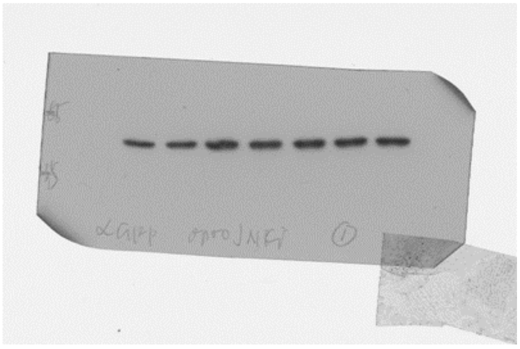

A. Whole length of gel shown corresponding to Fig. 2A
B. Whole film of blot corresponding to Fig. 2E

Supplementary Figure 3: Whole film lengths for images in Fig. 4

A

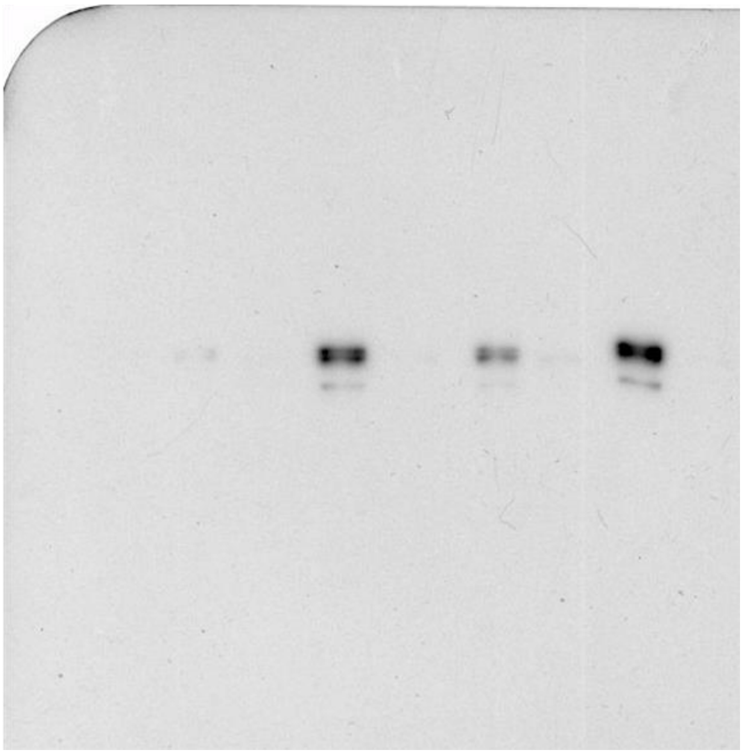

B.

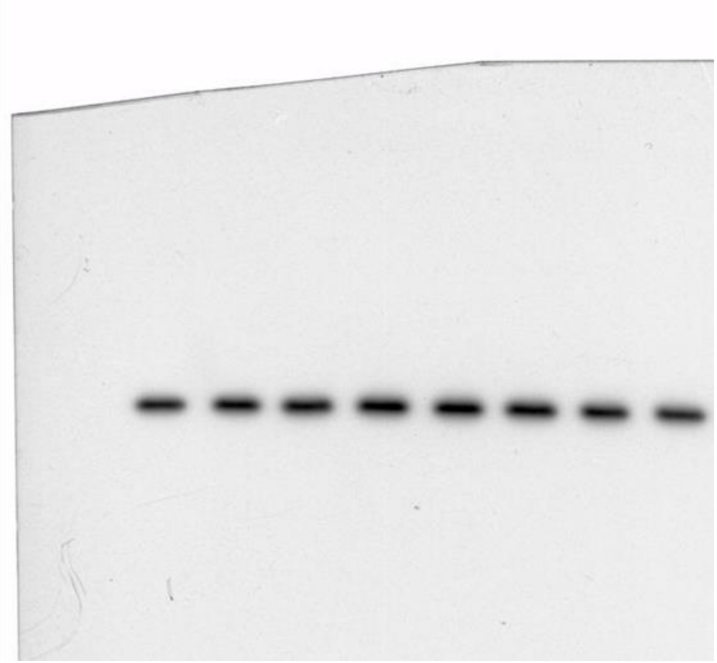

A. Film of whole length of blot shown in Fig. 5A (Phospho-Serine 73 c-Jun blot)

B. Film of whole length of blot shown in Fig. 5B (β-actin blot)

Supplementary Figure 4: Whole length of gels and films for Fig. 10

A.

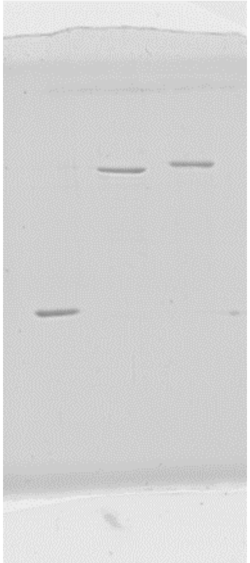

B.

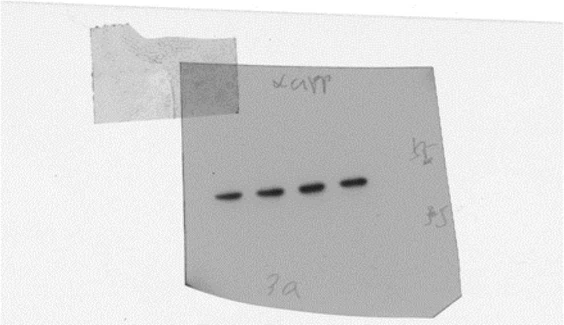

C.

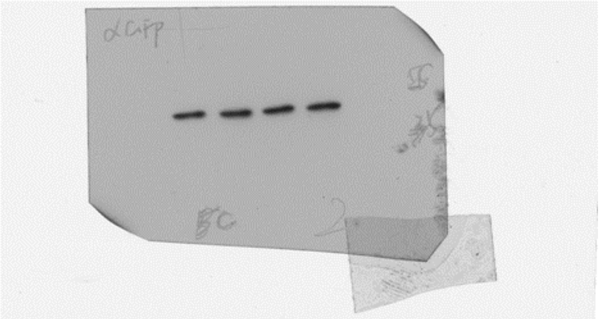

D.

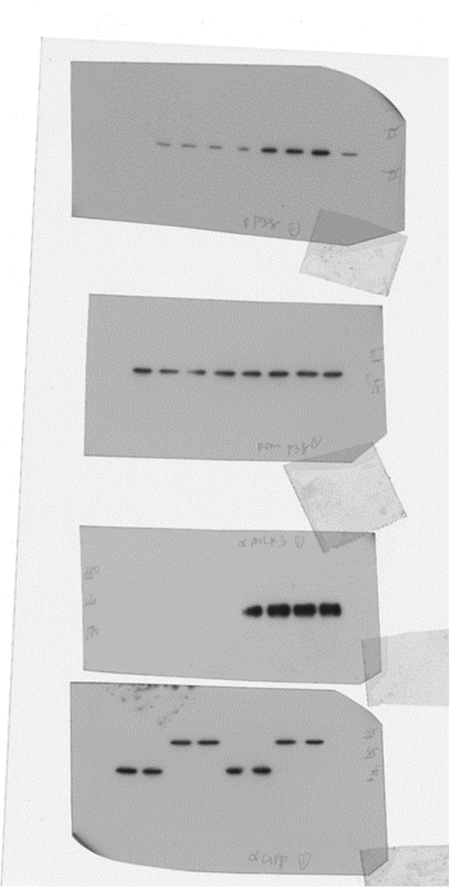

A. Whole length of gel corresponding to Fig. 10B. Lane 1 is GST, not shown in Fig. 10B.

B. Whole film corresponding to Fig. 10H, OptoJNKi3-13F.lsm

C. Whole film corresponding to Fig. 10H, OptoJNKi5-13F.dsm

D. Whole films corresponding to Fig. 10I, from top: phospho-p38, pan-p38, MLK3, GFP.

Supplementary Figure 5: Amino acid sequences of Optop38i sequences used with Clustal Omega[1] alignment.

```
>Optop38i(1-13F).dsm
LATTLERIEKNFVITDPRLPDNPIIFASDSFLQLTEYSREEILGRNARFLQGPETDRATVRKIRDAIDNQTEVTVQLINYTKSGKKFWNLFHLQPMRDQKGDVQYFI
GVQLDGTEHVRDAAEREGVMLIKKTAENIDEAAKELSRKSKRKKDLRISCMF*

>Optop38i(1-13F).lsm
LATTLERIEKNFVITDPRLPDNPIIFASDSFLQLTEYSREEILGRNCRFLQGPETDRATVRKIRDAIDNQTEVTVQLINYTKSGKKFWNLFHLQPMRDQKGDVQYFI
GVQLDGTEHVRDAAEREGVMLIKKTAENEDEAAKELSRKSKRKKDLRISCMF*

>Optop38i(1-13).dsm
LATTLERIEKNFVITDPRLPDNPIIFASDSFLQLTEYSREEILGRNARFLQGPETDRATVRKIRDAIDNQTEVTVQLINYTKSGKKFWNLFHLQPMRDQKGDVQYFI
GVQLDGTEHVRDAAEREGVMLIKKTAENIDEAAKELSRKSKRKKDLRISCM*

>Optop38i(1-13).lsm
LATTLERIEKNFVITDPRLPDNPIIFASDSFLQLTEYSREEILGRNCRFLQGPETDRATVRKIRDAIDNQTEVTVQLINYTKSGKKFWNLFHLQPMRDQKGDVQYFI
GVQLDGTEHVRDAAEREGVMLIKKTAENEDEAAKELSRKSKRKKDLRISCM*

>Optop38i(1-10F).dsm
LATTLERIEKNFVITDPRLPDNPIIFASDSFLQLTEYSREEILGRNARFLQGPETDRATVRKIRDAIDNQTEVTVQLINYTKSGKKFWNLFHLQPMRDQKGDVQYFI
GVQLDGTEHVRDAAEREGVMLIKKTAENIDEAAKELSRKSKRKKDLRIF*

>Optop38i(1-10F).lsm
LATTLERIEKNFVITDPRLPDNPIIFASDSFLQLTEYSREEILGRNCRFLQGPETDRATVRKIRDAIDNQTEVTVQLINYTKSGKKFWNLFHLQPMRDQKGDVQYFI
GVQLDGTEHVRDAAEREGVMLIKKTAENEDEAAKELSRKSKRKKDLRIF*

>Optop38i(2-13).dsm
LATTLERIEKNFVITDPRLPDNPIIFASDSFLQLTEYSREEILGRNARFLQGPETDRATVRKIRDAIDNQTEVTVQLINYTKSGKKFWNLFHLQPMRDQKGDVQYFI
GVQLDGTEHVRDAAEREGVMLIKKTAENIDEAAKELSRSKRKKDLRISCM*

>Optop38i(2-13).lsm
LATTLERIEKNFVITDPRLPDNPIIFASDSFLQLTEYSREEILGRNCRFLQGPETDRATVRKIRDAIDNQTEVTVQLINYTKSGKKFWNLFHLQPMRDQKGDVQYFI
GVQLDGTEHVRDAAEREGVMLIKKTAENEDEAAKELSRSKRKKDLRISCM*

>Optop38i(2-13F).dsm
LATTLERIEKNFVITDPRLPDNPIIFASDSFLQLTEYSREEILGRNARFLQGPETDRATVRKIRDAIDNQTEVTVQLINYTKSGKKFWNLFHLQPMRDQKGDVQYFI
GVQLDGTEHVRDAAEREGVMLIKKTAENIDEAAKELSRSKRKKDLRISCMF*

>Optop38i(2-13F).lsm
LATTLERIEKNFVITDPRLPDNPIIFASDSFLQLTEYSREEILGRNCRFLQGPETDRATVRKIRDAIDNQTEVTVQLINYTKSGKKFWNLFHLQPMRDQKGDVQYFI
GVQLDGTEHVRDAAEREGVMLIKKTAENEDEAAKELSRSKRKKDLRISCMF*

>Optop38i(3-13).dsm
LATTLERIEKNFVITDPRLPDNPIIFASDSFLQLTEYSREEILGRNARFLQGPETDRATVRKIRDAIDNQTEVTVQLINYTKSGKKFWNLFHLQPMRDQKGDVQYFI
GVQLDGTEHVRDAAEREGVMLIKKTAENIDEAAKELSRKRKKDLRISCM*

>Optop38i(3-13).lsm
LATTLERIEKNFVITDPRLPDNPIIFASDSFLQLTEYSREEILGRNCRFLQGPETDRATVRKIRDAIDNQTEVTVQLINYTKSGKKFWNLFHLQPMRDQKGDVQYFI
GVQLDGTEHVRDAAEREGVMLIKKTAENEDEAAKELSRKRKKDLRISCM*

>Optop38i(3-13F).dsm
LATTLERIEKNFVITDPRLPDNPIIFASDSFLQLTEYSREEILGRNARFLQGPETDRATVRKIRDAIDNQTEVTVQLINYTKSGKKFWNLFHLQPMRDQKGDVQYFI
GVQLDGTEHVRDAAEREGVMLIKKTAENIDEAAKELSRKRKKDLRISCMF*

>Optop38i(3-13F).lsm
LATTLERIEKNFVITDPRLPDNPIIFASDSFLQLTEYSREEILGRNCRFLQGPETDRATVRKIRDAIDNQTEVTVQLINYTKSGKKFWNLFHLQPMRDQKGDVQYFI
GVQLDGTEHVRDAAEREGVMLIKKTAENEDEAAKELSRKRKKDLRISCMF*

>Optop38i(4-13).dsm
LATTLERIEKNFVITDPRLPDNPIIFASDSFLQLTEYSREEILGRNARFLQGPETDRATVRKIRDAIDNQTEVTVQLINYTKSGKKFWNLFHLQPMRDQKGDVQYFI
GVQLDGTEHVRDAAEREGVMLIKKTAENIDEAAKELSRRKKDLRISCM*

>Optop38i(4-13).lsm
LATTLERIEKNFVITDPRLPDNPIIFASDSFLQLTEYSREEILGRNCRFLQGPETDRATVRKIRDAIDNQTEVTVQLINYTKSGKKFWNLFHLQPMRDQKGDVQYFI
GVQLDGTEHVRDAAEREGVMLIKKTAENEDEAAKELSRRKKDLRISCM*

>Optop38i(4-13F).dsm
LATTLERIEKNFVITDPRLPDNPIIFASDSFLQLTEYSREEILGRNARFLQGPETDRATVRKIRDAIDNQTEVTVQLINYTKSGKKFWNLFHLQPMRDQKGDVQYFI
GVQLDGTEHVRDAAEREGVMLIKKTAENIDEAAKELSRRKKDLRISCMF*

>Optop38i(4-13F).lsm
LATTLERIEKNFVITDPRLPDNPIIFASDSFLQLTEYSREEILGRNCRFLQGPETDRATVRKIRDAIDNQTEVTVQLINYTKSGKKFWNLFHLQPMRDQKGDVQYFI
GVQLDGTEHVRDAAEREGVMLIKKTAENEDEAAKELSRRKKDLRISCMF*

>Optop38i(5-13).dsm
LATTLERIEKNFVITDPRLPDNPIIFASDSFLQLTEYSREEILGRNARFLQGPETDRATVRKIRDAIDNQTEVTVQLINYTKSGKKFWNLFHLQPMRDQKGDVQYFI
GVQLDGTEHVRDAAEREGVMLIKKTAENIDEAAKELSRKKDLRISCM*

>Optop38i(5-13).lsm
LATTLERIEKNFVITDPRLPDNPIIFASDSFLQLTEYSREEILGRNCRFLQGPETDRATVRKIRDAIDNQTEVTVQLINYTKSGKKFWNLFHLQPMRDQKGDVQYFI
GVQLDGTEHVRDAAEREGVMLIKKTAENEDEAAKELSRKKDLRISCM*
```

>Optop38i(5-13F).dsm
LATTLERIEKNFVITDPRLPDNPIIFASDSFLQLTEYSREEILGRNARFLQGPETDRATVRKIRDAIDNQTEVTVQLINYTKSGKKFWNLFHLQPMRDQKGDVQYFI
GVQLDGTEHVRDAAEREGVMLIKKTAENIDEAAKELSRKKDLRISCMF*

>Optop38i(5-13F).lsm
LATTLERIEKNFVITDPRLPDNPIIFASDSFLQLTEYSREEILGRNCRFLQGPETDRATVRKIRDAIDNQTEVTVQLINYTKSGKKFWNLFHLQPMRDQKGDVQYFI
GVQLDGTEHVRDAAEREGVMLIKKTAENEDEAAKELSRKKDLRISCMF*

>Optop38i(5-13L).dsm
LATTLERIEKNFVITDPRLPDNPIIFASDSFLQLTEYSREEILGRNARFLQGPETDRATVRKIRDAIDNQTEVTVQLINYTKSGKKFWNLFHLQPMRDQKGDVQYFI
GVQLDGTEHVRDAAEREGVMLIKKTAENIDEAAKELSRKKDLRISCML*

>Optop38i(5-13L).lsm
LATTLERIEKNFVITDPRLPDNPIIFASDSFLQLTEYSREEILGRNCRFLQGPETDRATVRKIRDAIDNQTEVTVQLINYTKSGKKFWNLFHLQPMRDQKGDVQYFI
GVQLDGTEHVRDAAEREGVMLIKKTAENEDEAAKELSRKKDLRISCML*

>Optop38i(5-13V).dsm
LATTLERIEKNFVITDPRLPDNPIIFASDSFLQLTEYSREEILGRNARFLQGPETDRATVRKIRDAIDNQTEVTVQLINYTKSGKKFWNLFHLQPMRDQKGDVQYFI
GVQLDGTEHVRDAAEREGVMLIKKTAENIDEAAKELSRKKDLRISCMV*

>Optop38i(5-13V).lsm
LATTLERIEKNFVITDPRLPDNPIIFASDSFLQLTEYSREEILGRNCRFLQGPETDRATVRKIRDAIDNQTEVTVQLINYTKSGKKFWNLFHLQPMRDQKGDVQYFI
GVQLDGTEHVRDAAEREGVMLIKKTAENEDEAAKELSRKKDLRISCMV*

>Optop38i3(3-13F)
LATTLERIEKNFVITDPRLPDNPIIFASDSFLQLTEYSREEILGRNCRFLQGPETDRATVRKIRDAIDNQTEVTVQLINYTKSGKKFWNLFHLQPMRDQKGDVQYFI
GVQLDGTEHVRDAAEREGVMLIKKTAENIDEAAKELSRKRKKDLRISCMF*

```
CLUSTAL O(1.2.3) multiple sequence alignment

Optop38i(1-13F).dsm      LATTLERIEKNFVITDPRLPDNPIIFASDSFLQLTEYSREEILGRNARFLQGPETDRATV
Optop38i(1-13F).lsm      LATTLERIEKNFVITDPRLPDNPIIFASDSFLQLTEYSREEILGRNCRFLQGPETDRATV
Optop38i(1-13).dsm       LATTLERIEKNFVITDPRLPDNPIIFASDSFLQLTEYSREEILGRNARFLQGPETDRATV
Optop38i(1-13).lsm       LATTLERIEKNFVITDPRLPDNPIIFASDSFLQLTEYSREEILGRNCRFLQGPETDRATV
Optop38i(1-10F).dsm      LATTLERIEKNFVITDPRLPDNPIIFASDSFLQLTEYSREEILGRNARFLQGPETDRATV
Optop38i(1-10F).lsm      LATTLERIEKNFVITDPRLPDNPIIFASDSFLQLTEYSREEILGRNCRFLQGPETDRATV
Optop38i(2-13).dsm       LATTLERIEKNFVITDPRLPDNPIIFASDSFLQLTEYSREEILGRNARFLQGPETDRATV
Optop38i(2-13).lsm       LATTLERIEKNFVITDPRLPDNPIIFASDSFLQLTEYSREEILGRNCRFLQGPETDRATV
Optop38i(2-13F).dsm      LATTLERIEKNFVITDPRLPDNPIIFASDSFLQLTEYSREEILGRNARFLQGPETDRATV
Optop38i(2-13F).lsm      LATTLERIEKNFVITDPRLPDNPIIFASDSFLQLTEYSREEILGRNCRFLQGPETDRATV
Optop38i(3-13).dsm       LATTLERIEKNFVITDPRLPDNPIIFASDSFLQLTEYSREEILGRNARFLQGPETDRATV
Optop38i(3-13).lsm       LATTLERIEKNFVITDPRLPDNPIIFASDSFLQLTEYSREEILGRNCRFLQGPETDRATV
Optop38i(3-13F).dsm      LATTLERIEKNFVITDPRLPDNPIIFASDSFLQLTEYSREEILGRNARFLQGPETDRATV
Optop38i(3-13F).lsm      LATTLERIEKNFVITDPRLPDNPIIFASDSFLQLTEYSREEILGRNCRFLQGPETDRATV
Optop38i(4-13).dsm       LATTLERIEKNFVITDPRLPDNPIIFASDSFLQLTEYSREEILGRNARFLQGPETDRATV
Optop38i(4-13).lsm       LATTLERIEKNFVITDPRLPDNPIIFASDSFLQLTEYSREEILGRNCRFLQGPETDRATV
Optop38i(4-13F).dsm      LATTLERIEKNFVITDPRLPDNPIIFASDSFLQLTEYSREEILGRNARFLQGPETDRATV
Optop38i(4-13F).lsm      LATTLERIEKNFVITDPRLPDNPIIFASDSFLQLTEYSREEILGRNCRFLQGPETDRATV
Optop38i(5-13).dsm       LATTLERIEKNFVITDPRLPDNPIIFASDSFLQLTEYSREEILGRNARFLQGPETDRATV
Optop38i(5-13).lsm       LATTLERIEKNFVITDPRLPDNPIIFASDSFLQLTEYSREEILGRNCRFLQGPETDRATV
Optop38i(5-13F).dsm      LATTLERIEKNFVITDPRLPDNPIIFASDSFLQLTEYSREEILGRNARFLQGPETDRATV
Optop38i(5-13F).lsm      LATTLERIEKNFVITDPRLPDNPIIFASDSFLQLTEYSREEILGRNCRFLQGPETDRATV
Optop38i(5-13L).dsm      LATTLERIEKNFVITDPRLPDNPIIFASDSFLQLTEYSREEILGRNARFLQGPETDRATV
Optop38i(5-13L).lsm      LATTLERIEKNFVITDPRLPDNPIIFASDSFLQLTEYSREEILGRNCRFLQGPETDRATV
Optop38i(5-13V).dsm      LATTLERIEKNFVITDPRLPDNPIIFASDSFLQLTEYSREEILGRNARFLQGPETDRATV
Optop38i(5-13V).lsm      LATTLERIEKNFVITDPRLPDNPIIFASDSFLQLTEYSREEILGRNCRFLQGPETDRATV
Optop38i3 (3-13F)        LATTLERIEKNFVITDPRLPDNPIIFASDSFLQLTEYSREEILGRNCRFLQGPETDRATV
                         **********************************************.*************

Optop38i(1-13F).dsm      RKIRDAIDNQTEVTVQLINYTKSGKKFWNLFHLQPMRDQKGDVQYFIGVQLDGTEHVRDA
Optop38i(1-13F).lsm      RKIRDAIDNQTEVTVQLINYTKSGKKFWNLFHLQPMRDQKGDVQYFIGVQLDGTEHVRDA
Optop38i(1-13).dsm       RKIRDAIDNQTEVTVQLINYTKSGKKFWNLFHLQPMRDQKGDVQYFIGVQLDGTEHVRDA
Optop38i(1-13).lsm       RKIRDAIDNQTEVTVQLINYTKSGKKFWNLFHLQPMRDQKGDVQYFIGVQLDGTEHVRDA
Optop38i(1-10F).dsm      RKIRDAIDNQTEVTVQLINYTKSGKKFWNLFHLQPMRDQKGDVQYFIGVQLDGTEHVRDA
Optop38i(1-10F).lsm      RKIRDAIDNQTEVTVQLINYTKSGKKFWNLFHLQPMRDQKGDVQYFIGVQLDGTEHVRDA
Optop38i(2-13).dsm       RKIRDAIDNQTEVTVQLINYTKSGKKFWNLFHLQPMRDQKGDVQYFIGVQLDGTEHVRDA
Optop38i(2-13).lsm       RKIRDAIDNQTEVTVQLINYTKSGKKFWNLFHLQPMRDQKGDVQYFIGVQLDGTEHVRDA
Optop38i(2-13F).dsm      RKIRDAIDNQTEVTVQLINYTKSGKKFWNLFHLQPMRDQKGDVQYFIGVQLDGTEHVRDA
Optop38i(2-13F).lsm      RKIRDAIDNQTEVTVQLINYTKSGKKFWNLFHLQPMRDQKGDVQYFIGVQLDGTEHVRDA
Optop38i(3-13).dsm       RKIRDAIDNQTEVTVQLINYTKSGKKFWNLFHLQPMRDQKGDVQYFIGVQLDGTEHVRDA
Optop38i(3-13).lsm       RKIRDAIDNQTEVTVQLINYTKSGKKFWNLFHLQPMRDQKGDVQYFIGVQLDGTEHVRDA
Optop38i(3-13F).dsm      RKIRDAIDNQTEVTVQLINYTKSGKKFWNLFHLQPMRDQKGDVQYFIGVQLDGTEHVRDA
Optop38i(3-13F).lsm      RKIRDAIDNQTEVTVQLINYTKSGKKFWNLFHLQPMRDQKGDVQYFIGVQLDGTEHVRDA
Optop38i(4-13).dsm       RKIRDAIDNQTEVTVQLINYTKSGKKFWNLFHLQPMRDQKGDVQYFIGVQLDGTEHVRDA
Optop38i(4-13).lsm       RKIRDAIDNQTEVTVQLINYTKSGKKFWNLFHLQPMRDQKGDVQYFIGVQLDGTEHVRDA
Optop38i(4-13F).dsm      RKIRDAIDNQTEVTVQLINYTKSGKKFWNLFHLQPMRDQKGDVQYFIGVQLDGTEHVRDA
Optop38i(4-13F).lsm      RKIRDAIDNQTEVTVQLINYTKSGKKFWNLFHLQPMRDQKGDVQYFIGVQLDGTEHVRDA
Optop38i(5-13).dsm       RKIRDAIDNQTEVTVQLINYTKSGKKFWNLFHLQPMRDQKGDVQYFIGVQLDGTEHVRDA
Optop38i(5-13).lsm       RKIRDAIDNQTEVTVQLINYTKSGKKFWNLFHLQPMRDQKGDVQYFIGVQLDGTEHVRDA
Optop38i(5-13F).dsm      RKIRDAIDNQTEVTVQLINYTKSGKKFWNLFHLQPMRDQKGDVQYFIGVQLDGTEHVRDA
Optop38i(5-13F).lsm      RKIRDAIDNQTEVTVQLINYTKSGKKFWNLFHLQPMRDQKGDVQYFIGVQLDGTEHVRDA
Optop38i(5-13L).dsm      RKIRDAIDNQTEVTVQLINYTKSGKKFWNLFHLQPMRDQKGDVQYFIGVQLDGTEHVRDA
Optop38i(5-13L).lsm      RKIRDAIDNQTEVTVQLINYTKSGKKFWNLFHLQPMRDQKGDVQYFIGVQLDGTEHVRDA
Optop38i(5-13V).dsm      RKIRDAIDNQTEVTVQLINYTKSGKKFWNLFHLQPMRDQKGDVQYFIGVQLDGTEHVRDA
Optop38i(5-13V).lsm      RKIRDAIDNQTEVTVQLINYTKSGKKFWNLFHLQPMRDQKGDVQYFIGVQLDGTEHVRDA
Optop38i3 (3-13F)         RKIRDAIDNQTEVTVQLINYTKSGKKFWNLFHLQPMRDQKGDVQYFIGVQLDGTEHVRDA
                         ************************************************************

Optop38i(1-13F).dsm      AEREGVMLIKKTAENIDEAAKELSRKSKRKKDLRISCMF*
Optop38i(1-13F).lsm      AEREGVMLIKKTAENEDEAAKELSRKSKRKKDLRISCMF*
Optop38i(1-13).dsm       AEREGVMLIKKTAENIDEAAKELSRKSKRKKDLRISCM*-
Optop38i(1-13).lsm       AEREGVMLIKKTAENEDEAAKELSRKSKRKKDLRISCM*-
Optop38i(1-10F).dsm      AEREGVMLIKKTAENIDEAAKELSRKSKRKKDLRIF*---
Optop38i(1-10F).lsm      AEREGVMLIKKTAENEDEAAKELSRKSKRKKDLRIF*---
Optop38i(2-13).dsm       AEREGVMLIKKTAENIDEAAKELSR-SKRKKDLRISCM*-
Optop38i(2-13).lsm       AEREGVMLIKKTAENEDEAAKELSR-SKRKKDLRISCM*-
Optop38i(2-13F).dsm      AEREGVMLIKKTAENIDEAAKELSR-SKRKKDLRISCMF*
Optop38i(2-13F).lsm      AEREGVMLIKKTAENEDEAAKELSR-SKRKKDLRISCMF*
Optop38i(3-13).dsm       AEREGVMLIKKTAENIDEAAKELSR--KRKKDLRISCM*-
Optop38i(3-13).lsm       AEREGVMLIKKTAENEDEAAKELSR--KRKKDLRISCM*-
Optop38i(3-13F).dsm      AEREGVMLIKKTAENIDEAAKELSR--KRKKDLRISCMF*
Optop38i(3-13F).lsm      AEREGVMLIKKTAENEDEAAKELSR--KRKKDLRISCMF*
Optop38i(4-13).dsm       AEREGVMLIKKTAENIDEAAKELSR---RKKDLRISCM*-
Optop38i(4-13).lsm       AEREGVMLIKKTAENEDEAAKELSR---RKKDLRISCM*-
Optop38i(4-13F).dsm      AEREGVMLIKKTAENIDEAAKELSR---RKKDLRISCMF*
Optop38i(4-13F).lsm      AEREGVMLIKKTAENEDEAAKELSR---RKKDLRISCMF*
Optop38i(5-13).dsm       AEREGVMLIKKTAENIDEAAKELSR----KKDLRISCM*-
Optop38i(5-13).lsm       AEREGVMLIKKTAENEDEAAKELSR----KKDLRISCM*-
Optop38i(5-13F).dsm      AEREGVMLIKKTAENIDEAAKELSR----KKDLRISCMF*
Optop38i(5-13F).lsm      AEREGVMLIKKTAENEDEAAKELSR----KKDLRISCMF*
Optop38i(5-13L).dsm      AEREGVMLIKKTAENIDEAAKELSR----KKDLRISCML*
Optop38i(5-13L).lsm      AEREGVMLIKKTAENEDEAAKELSR----KKDLRISCML*
Optop38i(5-13V).dsm      AEREGVMLIKKTAENIDEAAKELSR----KKDLRISCMV*
Optop38i(5-13V).lsm      AEREGVMLIKKTAENEDEAAKELSR----KKDLRISCMV*
Optop38i3 (3-13F)        AEREGVMLIKKTAENIDEAAKELSR--KRKKDLRISCMF*
                         *************** *********    ******
```

Supplementary Figure 6: List of plasmids used in this study (plasmids in bold presented here for the first time)

pGEX-6P-hJNK1α1
pGEX-6P-hJNK2β1
pGEX-6P-hJNK3α1
pGEX-6P1
pGEX-hp38αtv2
pET28a-His-TAT-LOV2Jα
pET28a-His-TAT-OptoJNKi
pmCherry-hMKK3b-EE
pEBG-rΔMEKK1(1174-1493)
pEGFP-hMLK3
pYpet-C1
pLuc-C1
pLuc-mERK2
pLuc-hp38αtv2
pLuc-hJNK1α1
pLuc-hJNK1β1
pLuc-hJNK2α2
pLuc-hJNK3α1
pLuc-hMKK3b
pCGN-GAL4-Mef2A
pcDNA3-GAL4-c-Jun(5-105)
pRL-CMV
pGL3-G5E4Δ38
pCMV
pH2B-Venus
pH2B-mCherry
pLifeact-Ypet
pmKeima620-NES-JIP1-277
pmKeima620-3xNLS-JIP1-277
pLuc-JIP1-277
pLuc-AsLOV2Jα-JIP10.lsm
pLuc-AsLOV2Jα-JIP10.dsm
pLuc-AsLOV2Jα-JIP11.lsm (OptoJNKi.lsm)
pLuc-AsLOV2Jα-JIP11.dsm (OptoJNKi.dsm)
pLuc-AsLOV2Jα(F509R)-JIP11.lsm (OptoJNKi(F509R).lsm)
pLuc-AsLOV2Jα(F509R)-JIP11.dsm (OptoJNKi(F509R).dsm)
pYpet-AsLOV2Jα-JIP11.dsm (Ypet-OptoJNKi.lsm)
pYpet-AsLOV2Jα-JIP11.lsm (Ypet-OptoJNKi.dsm)
pLuc-OptoJNKi
pmCherry-NLS-OptoJNKi
pmCherry-NLS-OptoJNKi.lsm
pmCherry-NES-OptoJNKi
pH2B-pmCherry-OptoJNKi
pmCherry-NLS-OptoJNKi.dsm
pLuc-AsLOV2Jα-JIP12.lsm
pLuc-AsLOV2Jα-JIP12.dsm
pLuc-AsLOV2Jα-JIP13.lsm
pLuc-AsLOV2Jα-JIP13.dsm
pLuc-Optop38i.1-13F.lsm

pLuc-Optop38i.1-13F.dsm
pLuc-Optop38i.1-10F.lsm
pLuc-Optop38i.1-10F.dsm
pLuc-Optop38i.1-13.lsm
pLuc-Optop38i.1-13.dsm
pLuc-Optop38i.2-13.lsm
pLuc-Optop38i.2-13.dsm
pLuc-Optop38i.2-13F.lsm
pLuc-Optop38i.2-13F.dsm
pLuc-Optop38i.3-13.lsm
pLuc-Optop38i.3-13.dsm
pLuc-Optop38i.3-13F.lsm
pLuc-Optop38i.3-13F.dsm
pYpet-Optop38i.3-13F.lsm
pLuc-Optop38i3 (LOV2Jα.3-13F)
pLuc-Optop38i.4-13.lsm
pLuc-Optop38i.4-13.dsm
pLuc-Optop38i.4-13F.lsm
pLuc-Optop38i.4-13F.dsm
pLuc-Optop38i.5-13.lsm
pLuc-Optop38i.5-13.dsm
pLuc-Optop38i.5-13F.lsm
pLuc-Optop38i.5-13F.dsm
pLuc-Optop38i.5-13L.lsm
pLuc-Optop38i.5-13L.dsm
pLuc-Optop38i.5-13V.lsm
pLuc-Optop38i.5-13V.dsm
pYpet-Optop38i.5-13F.dsm

Supplementary References

1. Sievers F, Wilm A, Dineen DG, Gibson TJ, Karplus K, Li W, Lopez R, McWilliam H, Remmert M, Söding J, Thompson JD, Higgins D (2011) Fast, scalable generation of high-quality protein multiple sequence alignments using Clustal Omega Molecular Systems Biology (2011) 7, 539.
